# A novel deep learning prediction method for PM2.5 integrating improved MSTL decomposition and FATA optimization algorithm

**DOI:** 10.1371/journal.pone.0358271

**Published:** 2026-09-15

**Authors:** Xiaoliang Zhao, Pinyuan Qiao, Bandna Bharti, Hanliang Li, Shitong Yang, Qi Shi, Qiuhong Qin, Jundian Chen

**Affiliations:** 1 Department of Environmental Science and Engineering, Liaoning Technical University, Fuxin, Liaoning, China; 2 Collaborative Innovation Center of Mine Major Disaster Prevention and Environmental Restoration, Fuxin, Liaoning, China; 3 Department of Chemistry, DAV University, Jalandhar, Punjab, India; 4 Inner Mongolia Shendong Tianlong Group Co., Ltd., Ordos, Inner Mongolia, China; 5 Liaoning Geo-Mineral Investment and Development Group Co., Ltd., Shenyang, Liaoning, China; 6 Liaoning Provincial Geology and Mineral Resources Group Mining Co., Ltd., Shenyang, Liaoning, China; National Chung Hsing University, TAIWAN

## Abstract

Accurate prediction of PM2.5 time series remains challenging. To address this issue, this paper proposes a novel PM2.5 concentration prediction model named FATA-GSPMSTL-CNN-LSTM.Firstly,the optimal feature descriptor set is determined by combining Recursive Feature Elimination with Cross-Validation (RFECV) and Pearson correlation analysis. On the basis of Multiple Seasonal-Trend decomposition using Loess (MSTL), a novel adaptive decomposition algorithm—Grid Search-based Power Spectral MSTL (GSPMSTL)—is constructed by introducing spectral density analysis and grid search strategy, which is adopted to decompose the PM2.5 time series, and the dataset is reconstructed through feature data fusion. The newly developed Fata morgana optimization algorithm (FATA) is utilized to optimize the model hyperparameters for further improving prediction accuracy. Finally, the Convolutional Neural Network- Long Short-Term Memory network (CNN-LSTM) is employed to obtain the final prediction results. Considering climate, topography and seasonal factors, PM2.5 prediction and evaluation are separately conducted in heating seasons and non-heating seasons for Guangzhou and Xianyang cities. The results demonstrate that the proposed model achieves higher prediction accuracy and stability, which can provide important application value for air quality early warning and pollution control.

## Introduction

Against the backdrop of global industrialization and rapid economic development, atmospheric pollution has become an increasingly prominent issue, posing severe challenges to environmental governance and public health protection [1]. As a core indicator for atmospheric pollution and health-risk assessment, PM2.5 is characterized by small particle size, long atmospheric residence time, and long-range transport capacity, acting as the primary driving factor for haze formation [2,3]. Existing studies have confirmed a significant positive correlation between PM2.5 concentrations and the incidence of hypertension [4,5]. Furthermore, PM2.5 can trigger human inflammatory responses. It not only damages vascular endothelial tissues but also substantially increases the prevalence of respiratory diseases, thereby constituting a serious threat to human health and safety [6–8]. Accordingly, accurate prediction of PM2.5 concentrations is of far-reaching significance for the effective implementation of atmospheric pollution prevention and control [9]. At present, numerous studies on PM2.5 concentration prediction have been conducted by scholars worldwide. According to their modeling mechanisms, these models can be classified into three categories: physical-mechanism models, statistical-analysis models, and intelligent prediction models [10–12].

Physical-mechanism models simulate the diffusion and sedimentation processes of atmospheric pollutants by constructing mathematical formulations based on the physicochemical principles governing pollutant generation, transport and deposition, mainly including the Weather Research and Forecasting model (WRF) and Community Multiscale Air Quality model (CMAQ) [13]. For instance, Jena et al. realized accurate prediction of PM2.5 concentrations over India during the winter of 2020 using the WRF-SLAM model [14]. Mohd Napi et al. performed sensitivity analysis of PM_2.5_ concentrations across the Malay Peninsula with the WRF-CMAQ model [15]. Schaap et al. systematically estimated anthropogenic aerosol concentrations over European regions using the LOTOS model [16]. Although such models can reasonably characterize the migration and transformation patterns of atmospheric pollutants under cross-regional pollution scenarios, the initialization of emission inventories and physicochemical parameters heavily relies on empirical assignment. Moreover, they incur substantial computational overhead for high-spatiotemporal- resolution prediction tasks.

Statistical-analysis models implement prediction by mining distribution patterns embedded in historical time-series data via probabilistic statistical approaches, including Autoregressive Moving Average (ARIMA), Principal Component Regression (PCR), Multiple Linear Regression (MLR), and other models [17]. Featuring simple structures and high interpretability, statistical-analysis models have relatively low computational costs for prediction with small-scale datasets and are therefore widely adopted for PM2.5 concentration prediction. For example, Chumnaul and Damkliang achieved high-accuracy PM2.5 forecasting for Hat Yai, Thailand, and conducted interpretability analysis on prediction outputs by integrating quantile regression, ARIMA and artificial neural networks (ANN) [18]. Wabinyai et al. constructed a modified multiple linear regression model to effectively predict PM2.5 concentrations in Kampala and Fort Portal, Uganda [19]. Nevertheless, these models exhibit limited capacity to extract high-dimensional feature correlations within time-series sequences and yield relatively low fitting accuracy for large-scale time-series prediction.

Constrained by spatiotemporal costs, both physical-mechanism and statistical-analysis models have encountered developmental bottlenecks. With the rapid advancement of computer technologies, intelligent prediction models have become a research hotspot in the field of PM2.5 prediction owing to their powerful nonlinear fitting capability and relatively low spatiotemporal prediction costs. Intelligent prediction models can be further divided into basic prediction models and hybrid-ensemble models. Basic prediction models refer to PM2.5 time-series forecasting models that adopt native network architectures without auxiliary modeling mechanisms, mainly including LSTM, GRU and other related models. For example, Li et al. obtained accurate hourly-scale PM2.5 concentration predictions for Beijing using an LSTM model, achieving a mean absolute percentage error (*MAPE*) as low as 11.93% [20]. Attanayake et al. proposed an RF-CNN hybrid model for spatiotemporal PM2.5 concentration modeling and prediction across Sri Lanka. This model yielded a mean absolute percentage error of 22.8% and maintained stable prediction performance across diverse climate zones [21]. Pan predicted hourly PM2.5 concentrations in Tianjin using XGBoost, and the results demonstrated that XGBoost outperformed other baseline models in prediction performance [22]. Kim et al. constructed a CNN-LSTM network to predict daily-scale PM2.5 time-series across South Korea [23]. However, such models have inherent upper limits in capturing complex nonlinear patterns embedded in time-series data, which restricts further improvement of prediction accuracy. Meanwhile, most existing relevant studies determine optimal hyperparameter combinations via empirical methods or orthogonal experiments, which easily traps models into local optima and impairs generalization performance across diverse prediction scenarios. Hybrid-ensemble models indirectly improve prediction accuracy by introducing additional auxiliary modeling strategies, which can be subdivided into deep-ensemble prediction models, heuristic-driven prediction models, and decomposition-driven prediction models. Deep-ensemble prediction models enhance inter-module coupling and collaborative optimization by introducing multiple heterogeneous components or predictors to boost model prediction performance. For example, Hossen et al. constructed four models (CCCFC, TRCFC, FLC and BLC) based on hourly PM2.5 feature data from 18 monitoring stations in Taipei. CCCFC and TRCFC adopt coupled closed-form continuous-time network architectures to enable continuous dynamic modeling. The results revealed that these four models exhibited distinctly superior prediction accuracy compared with conventional baseline models, alongside robust performance stability across full-time periods and multiple monitoring stations [24,25]. Sun and Li built a two-layer Stacking-driven ensemble model for hourly PM2.5 concentration prediction over the Beijing-Tianjin-Hebei region in winter. The proposed model enabled precise prediction for highly complex PM2.5 time-series with extremely high concentrations [26]. Heuristic-driven prediction models improve model performance by introducing diverse hyperparameter optimization strategies to identify optimal hyperparameter combinations. Common meta-heuristic optimization algorithms include particle swarm optimization (PSO), sparrow search optimization algorithm (SSA), whale optimization algorithm (WOA) and so on. For example, Zandi et al. proposed a hybrid prediction model combining echo state network and SSA optimization (ESN-SSA). This model achieved remarkably superior prediction performance over competing models for PM2.5 prediction in Tehran, Iran [27]. Wei et al. developed a WOA-CNN-LSTM-AM hybrid prediction model for daily PM2.5 forecasting in Beijing, which produced smaller prediction errors than the standalone LSTM model [28]. Nevertheless, to pursue higher optimization precision, meta-heuristic algorithms adopted in some existing studies generally suffer from slow convergence rates, leading to high time costs during model training. Decomposition-driven prediction models fuse modal decomposition algorithms with intelligent prediction models, where decomposed modal components are fed as input features for subsequent time-series modeling. Representative modal decomposition algorithms include seasonal-trend decomposition (STL), empirical mode decomposition (EMD), complete ensemble empirical mode decomposition with adaptive noise (CEEMDAN), and variational mode decomposition (VMD) [29,30]. Modal decomposition effectively mitigates the burden of mining complex nonlinear evolutionary patterns within time-series data and thus substantially improves prediction accuracy, which has contributed to its widespread application. For instance, Teng et al. proposed a CEEMD-AE-BiLSTM model and conducted comparative experiments using data from multiple monitoring stations in Beijing, verifying that the target model achieved lower prediction errors [31]. Wang et al. integrated EMD with LSTM, SVM and ARIMA to construct a Hybrid-EMDHL deep-learning model for hourly-scale PM2.5 prediction across six core cities in North China. The hybrid model yielded a direction-accuracy (*DA*) metric above 0.69 and outperformed comparative models in predicting time-series evolutionary trends [32]. In addition, benefiting from favorable decomposition performance, fast computation speed and interpretable components, the STL decomposition algorithm has been adopted in various time-series forecasting tasks and achieved promising outcomes [33,34]. Despite the generally competitive prediction performance of decomposition-driven models, their decomposition strategies still possess considerable room for improvement in terms of adaptability across diverse time-series prediction scenarios. Specifically, initialization parameters for modal decomposition are mostly determined empirically, resulting in substantial uncertainty in decomposition quality under different task conditions, which severely limits further improvement of generalization performance for multi-scenario prediction.

From the perspective of feature engineering, existing studies on PM2.5 concentration prediction can be categorized into single-feature modeling strategies and multi-feature modeling strategies. Multi-feature modeling strategies incorporate multiple influencing factors such as meteorological conditions and pollution sources, supplying models with complex coupling associations among diverse features and thus exhibiting advantages in prediction accuracy and generalization capability. However, most existing multi-feature modeling studies adopt a single feature-screening strategy to identify feature importance; for example quantifying feature importance via Pearson or Kendall correlation analysis for feature selection. Such approaches can only mine feature information from a single correlation dimension, cannot sufficiently characterize complex linear and nonlinear coupling relationships among features, and thus yield biased feature-evaluation outcomes [35–38].

In summary, existing decomposition-driven hybrid models suffer from deficiencies in the adaptability and interpretability of decomposition algorithms, and effective multi-scale feature-screening strategies are lacking to refine current PM2.5 prediction frameworks. Additionally, for research on heuristic-driven prediction models, novel hyperparameter optimization algorithms are urgently required to achieve a favorable trade-off between convergence speed and optimization precision. To address the above-mentioned issues, this study first combines recursive feature elimination with cross-validation (RFECV) and Pearson correlation analysis and proposes an RFECV-Pearson feature-screening strategy. Feature importance is accurately quantified from both linear and nonlinear correlation perspectives to preliminarily identify the optimal set of feature descriptors for each city. Subsequently, this study introduces power spectral density analysis and grid-search optimization to modify the MSTL decomposition algorithm, and proposes an improved multiple seasonal-trend decomposition algorithm named GSPMSTL. Taking each city as a unit, this study conducts mode decomposition for PM2.5 time-series features. The decomposed trend term, several seasonal sub-terms and residual term are adopted as auxiliary features to expand the set of feature descriptors of the dataset, and the finally reconstructed dataset is applied to the subsequent time-series modeling process. Based on the above strategies, we aim to effectively improve the adaptive capability of the MSTL decomposition algorithm under multi-scenario prediction tasks as well as the model’s capability to mine complex time-series patterns. Meanwhile, the FATA is employed for hyperparameter tuning of the CNN-LSTM hybrid model to realize a favorable balance between PM2.5 prediction accuracy and convergence speed. By combining these above strategies, a novel hybrid-ensemble PM2.5 prediction model named FATA-GSPMSTL-CNN-LSTM is constructed. Experimental results demonstrate that the target model outperforms other baseline models in terms of PM2.5 prediction accuracy and stability, and can provide critical technical support for atmospheric pollution early-warning and prevention-control practices.

The core innovations of this study are summarized as follows:

1 An RFECV-Pearson feature-screening strategy is proposed to determine the optimal feature descriptor set and accomplish feature selection.
2 The MSTL decomposition algorithm is improved using power spectral density analysis and grid-search strategies, and a modified multiple seasonal-trend decomposition algorithm termed GSPMSTL is proposed to assist in boosting the prediction accuracy of deep-learning models.
3 FATA is introduced to determine the optimal hyperparameter combination for the CNN-LSTM hybrid model, achieving a favorable balance between convergence speed and convergence precision.
4 Multiple groups of comparative experiments are implemented to fully verify the performance superiority of the FATA-GSPMSTL-CNN-LSTM hybrid model over reference models in PM2.5 prediction, providing new insights for follow-up relevant research.

To ensure consistent usage of abbreviations throughout the paper, the names and corresponding abbreviations of key terms in this study are listed in Table 1.

**Table 1:** Name Abbreviations.

Abbreviation	Description	Abbreviation	Description
EMD	Empirical Mode Decomposition	M1	QRGRU
VMD	Variational Mode Decomposition	M2	TimesNet
CEEMDAN	Complete Ensemble Empirical Mode Decomposition with Adaptive Noise	M3	DLinear
MSTL	Multiple Seasonal-Trend Decomposition on using Loess	M4	LSTM
GSPMSTL	An improved MSTL algorithm based on grid search and spectral density analysis	M5	CNN-LSTM
SSA	Sparrow Search Optimization Algorithm	M6	EMD-CNN-LSTM
WOA	Whale Optimization Algorithm	M7	VMD-CNN-LSTM
FATA	Fata Morgana Optimization Algorithm	M8	FATA-GSPMSTL -CNN-LSTM

## Materials and methods

### Research area

**Fig 1:**
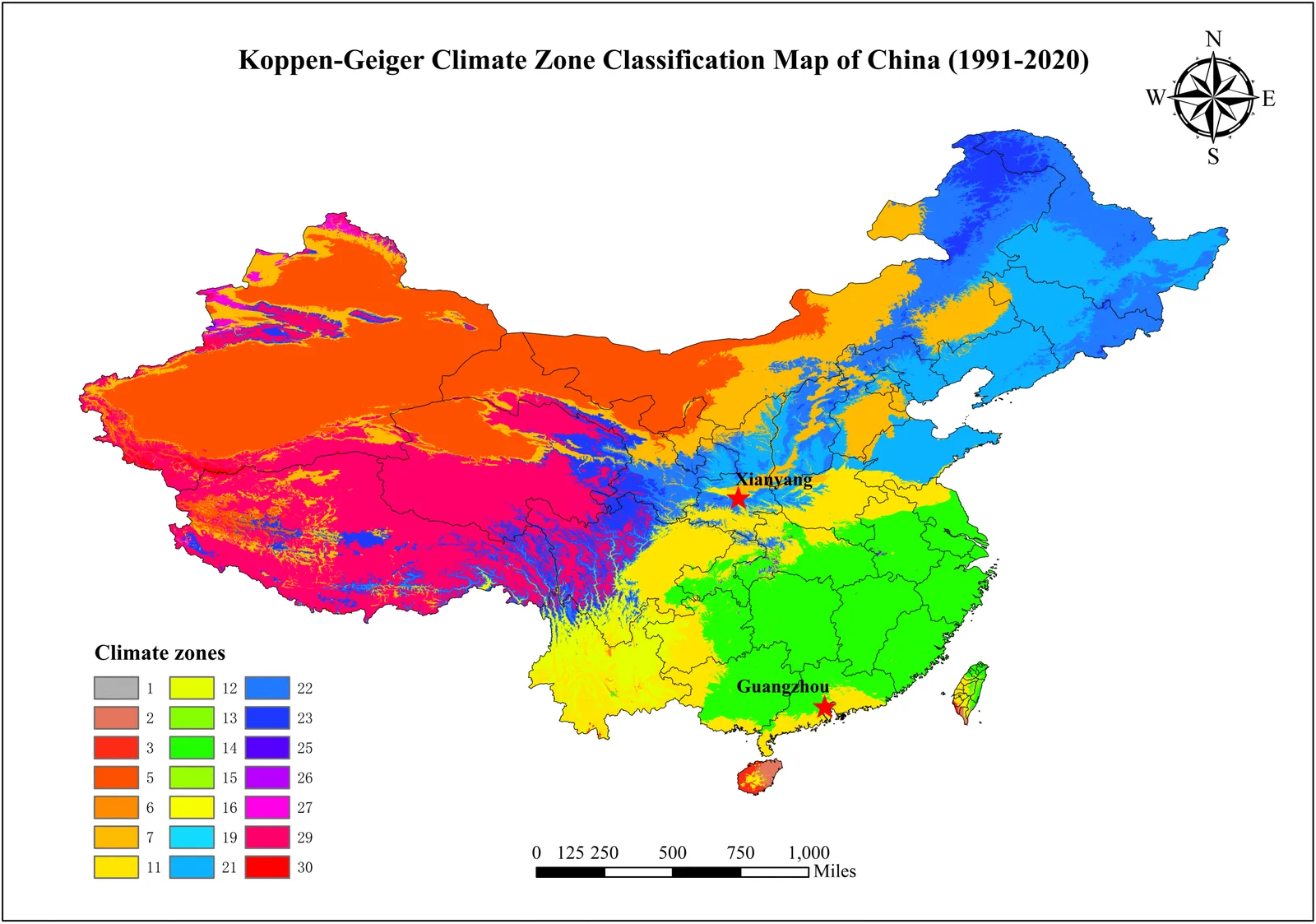
Climate zone division of China.

To effectively verify the generalization performance of the model, according to the Köppen-Geiger climate classification system [39], this paper selects Xianyang, the representative city of temperate continental monsoon climate (Dwa), and Guangzhou, the representative city of humid subtropical climate (Cfa), as the target study areas (as shown in Fig 1). Located in the Guanzhong Basin, Xianyang is an important energy-supply region in central-western China. Situated at the core of the Pearl River Delta, Guangzhou features intensive industrial activities and strong population mobility, and serves as an economic core zone along China’s southern coast. There exist remarkable differences between the two cities in terms of climate, topography, pollutant emissions and meteorological diffusion conditions. Therefore, selecting these two cities for research related to PM2.5 prediction carries important reference value.

### Data sources and preprocessing

The PM2.5 feature dataset adopted in this study mainly consists of two parts: pollutant concentration data and meteorological data. Among them, the pollutant concentration data are derived from the Chinese Earth System Science Data Center, specifically covering six types of pollutants including SO2, NO2, CO, O3, PM2.5 and PM10. The meteorological data are obtained from the European Centre for Medium-Range Weather Forecasts (ECMWF), specifically including 2-meter air temperature (t2m), 2-meter dew-point temperature (d2m), 10-meter U-component of wind (u10), 10-meter V-component of wind (v10), and 550 nm aerosol optical depth (AOD). The time span of the dataset ranges from 00:00 on January 1, 2020–23:00 on December 31, 2024. The pollutant data are sampled at an hourly scale, whereas the meteorological data are sampled at a daily scale, yielding a total of 43848 valid observation records. To avoid data leakage in the subsequent data preprocessing stage, we first perform dataset partitioning: the entire dataset is split into a training set and a test set at a 4:1 ratio, and the training set is further divided into a training subset and a validation subset at a 3:1 ratio. Afterwards, data preprocessing operations including missing-value processing, outlier processing and normalization are independently implemented on each divided data subset in sequence.

Specifically, a time-series-based interpolation method is employed for missing-value filling, and its specific calculation principle is shown in Eq. (1):


$$\begin{array}{c} y\;=\;y_{\text{1}}\;+\;\frac{y_{\text{2}}\;{-\;y}_{\text{1}}}{t_{\text{2}}{\;-\;t}_{\text{1}}}\;\times \;(t\;-\;t_{\text{1}}) \end{array}$$ (1)

where *y* denotes the filled value; *t* is the time corresponding to the missing value; *t*_1_ and *t*_2_ represent the time instant immediately before and after *t* respectively; *y*_1_ and *y*_2_ are the actual values at time *t*_1_ and *t*_2_.

We further adopt the quartile method to identify and process outliers. This strategy determines the upper and lower bounds of the normal fluctuation interval of the time-series data by calculating the first quartile (*Q*_1_), the third quartile (*Q*_3_) and their difference (*IQR*) of the time series. On this basis, we further carry out the identification and processing of outliers: all data points below the lower bound or above the upper bound are regarded as outliers and eliminated; conversely, those within the bounds are treated as normal values and retained. Meanwhile, these vacant values are further filled by calculating the mode of the time series, and the specific calculation is shown in Eq. (2).


$$\begin{array}{c} \left\{ \begin{array}{c} IQR\;=\;Q_{\text{3}}\;-\;Q_{\text{1}}\;\\ LowerBound\;=\;Q_{\text{1}}\;-\;\text{1}.\text{5}\;\times \;IQR\\ UpperBound\;={\;Q}_{\text{3}}\;+\;\text{1}.\text{5}\;\times \;IQR \end{array}\right. \end{array}$$ (2)

where *IQR* denotes the interquartile range; *Q*_1_ is the first quartile of the time-series dataset; *Q*_3_ represents the third quartile of the time-series dataset; *LowerBound* is the lower bound for normal data fluctuation; *UpperBound* stands for the upper bound for normal data fluctuation.

### Chaotic characteristic analysis

Chaotic dynamic characteristics mean that a time series is generated by a deterministic nonlinear system and satisfies sensitive dependence on initial conditions, topological transitivity and density of periodic orbits, thus presenting complex evolutionary behaviors that appear to fluctuate randomly on the surface yet contain deterministic laws internally. Chaotic characteristic analysis refers to a scientific method that systematically characterizes the chaotic properties of time series by adopting quantitative indicators including Kolmogorov entropy, sample entropy and Lyapunov exponent. This strategy can not only provide theoretical interpretability for the introduction of modal decomposition strategies, but also deliver critical data support for the adaptive modification of decomposition algorithms. Therefore, we select Kolmogorov entropy and sample entropy as quantitative indicators of chaotic characteristics to conduct chaotic characteristic analysis on the respective PM2.5 time series of Guangzhou and Xianyang.

### RFECV-Pearson feature analysis

RFECV is a feature selection method that combines a greedy search algorithm with a cross-validation mechanism, whose core objective is to select the optimal feature subset from the set of feature descriptors to avoid feature redundancy. The Pearson feature analysis method is a feature analysis approach for quantifying the linear correlation degree between two feature variables. In this study, RFECV and Pearson are used to realize collaborative feature screening to ensure the rationality of the construction of the feature descriptor set.

### Improved multiple seasonal-trend decomposition strategy

#### Spectral density analysis method.

Spectral density analysis is a commonly used period identification method in time series analysis. It mines the potential periodic laws of time series by decomposing complex time series into harmonic components of different frequencies [40]. Spectral density analysis methods include the periodogram method, Welch method, etc. Among them, the periodogram method can intuitively present the energy intensity of each frequency through an energy-frequency graph, and can quickly identify the main periodic characteristics of time series. In this study, we perform power spectral density analysis on the PM2.5 time series under the prediction scenarios of different cities based on the periodogram method, and adaptively determine part of the initialization parameters for GSPMSTL decomposition by identifying the dominant periods contained in the time series.

#### GSPMSTL decomposition strategy.

MSTL is an improved algorithm based on the STL single seasonal decomposition strategy. Based on Loess smoothing technology, this decomposition method can decompose a time series into one trend term, multiple seasonal terms and one residual term, which can effectively capture multiple seasonal periodic laws and is suitable for processing time series with multi-seasonal periodic characteristics [41]. The detailed pseudocode of the MSTL decomposition algorithm is shown in S1 Table, and the decomposition formulas of STL and MSTL are shown in Eqs. (3) and (4) respectively:


$$\begin{array}{c} X_{t\;}=\;T_{t}\;+\;S_{t}\;+{\;R}_{t\;} \end{array}$$ (3)

$$\begin{array}{c} X_{t}\;={\;T}_{t}\;+\sum_{i=1}^{k}S_{t}^{i}+\;R_{t} \end{array}$$ (4)

where *X*_*t*_ is the observed value of the time series at time *t*; *T*_*t*_ is the decomposed value of the trend term at time *t*; *S*_*t*_ is the decomposed value of the seasonal term at time *t*; $S_{t}^{i}$is the decomposed value of the *i*-th seasonal term obtained by MSTL decomposition at time *t*; *R*_*t*_ is the decomposed value of the residual term at time *t*. Although the MSTL decomposition algorithm yields highly interpretable decomposition results and can achieve high-accuracy decomposition for complex time series containing multiple periodic features, multiple factors still restrict its practical application in time-series modeling tasks. First, MSTL decomposition performs time-step-by-time-step smoothing-fitting computation for time series using a centrally-symmetric local-weighted regression smoothing window. This means that when solving the smoothed value at the current time step, the algorithm relies on the values of future-time-step data, which causes forward-looking data leakage. Second, the period-factor parameters of MSTL are mainly determined by empirical methods, and the supporting seasonal-smoothing-window parameters are calculated via a fixed-mapping mechanism. Considering that the initialization status of MSTL decomposition parameters significantly affects the quality of decomposition outputs, this severely limits the improvement of the adaptive capability and generalization performance of the MSTL decomposition algorithm across diverse application scenarios.

**Fig 2:**
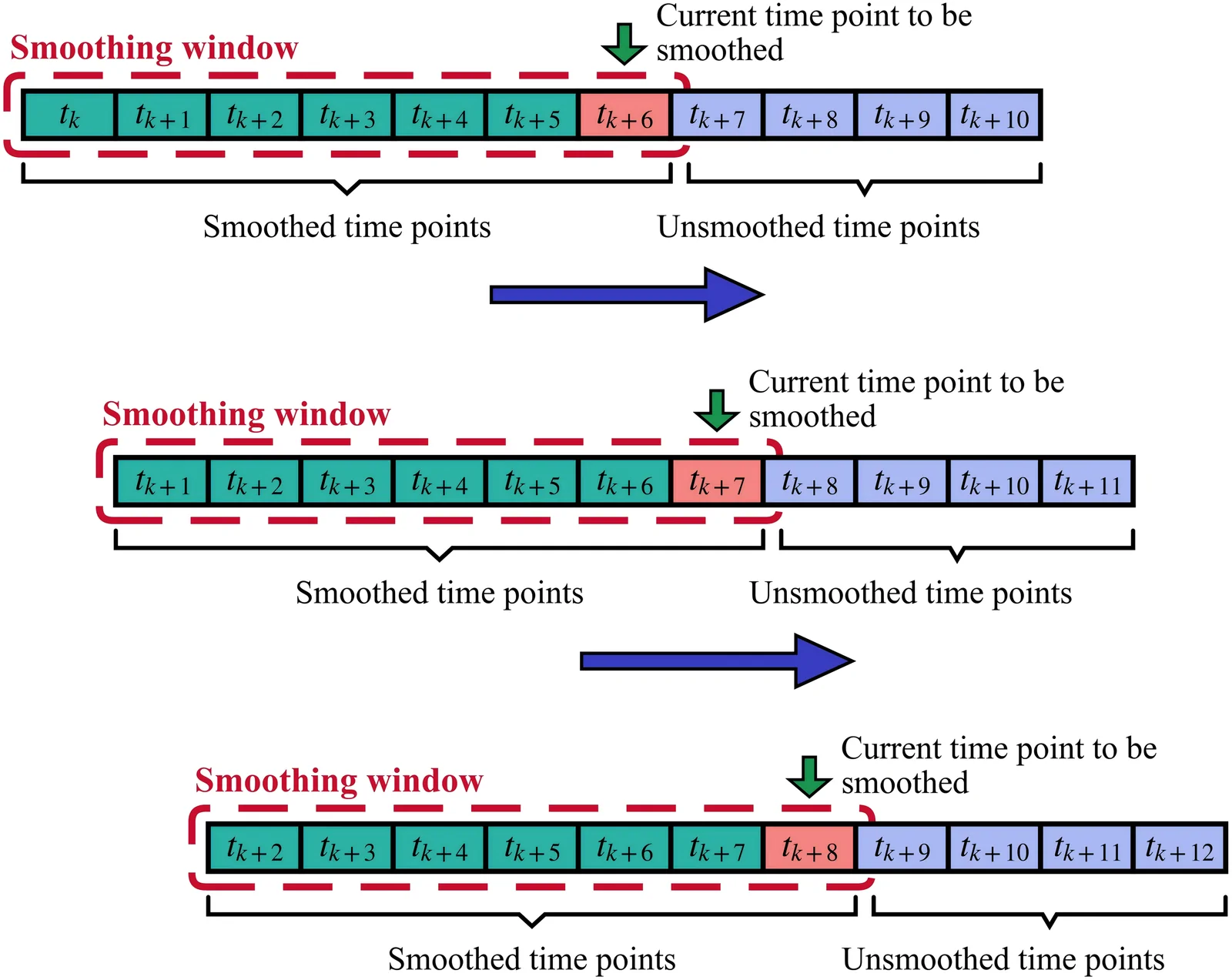
Computational logic of the left-biased asymmetric smoothing mechanism.

To effectively solve the above-mentioned problems, we make appropriate modifications to the MSTL decomposition algorithm and propose an improved multi-seasonal-trend decomposition algorithm named GSPMSTL. Specifically, to prevent the decomposition algorithm from suffering from data leakage, we introduce a left-biased asymmetric smoothing window to replace the centrally-symmetric smoothing window, and optimize the local-weighted-regression smoothing computation procedure of MSTL decomposition. The logic of the improved smoothing computation is illustrated in Fig 2 (assuming a smoothing-window size of 7 in the figure). It can be observed from the figure that under the left-biased asymmetric smoothing mechanism, the calculation of the smoothed value at each time step relies solely on the time-series values of the current and historical time points. Compared with the centrally-symmetric smoothing mechanism, this approach effectively prevents future-time-step data from being involved in the smoothing computation process, so that the improved decomposition algorithm is free from forward-looking data leakage.

Firstly, this algorithm adopts the periodogram-based power-spectral- density-analysis strategy to adaptively identify the dominant periods of the PM2.5 time series, which are taken as the initialized seasonal-period-factor parameters of the MSTL decomposition algorithm. On this basis, we select the Seasonal Separation Degree (*SSD*) as the objective optimization index, adopt the grid-search optimization strategy to determine the corresponding optimal seasonal-smoothing-window value for each period factor, and further validate the optimization results using the *Fuzzy_Entropy_Sum* index. Both indices serve as critical metrics for evaluating the decomposition quality of the MSTL algorithm: *SSD* reflects the correlation degree among various seasonal components; a smaller *SSD* value indicates weaker correlation between different seasonal components and better separation performance of seasonal components. *Fuzzy_Entropy_Sum* reflects the overall complexity of each decomposed component. A smaller *Fuzzy_Entropy_Sum* value denotes lower random volatility and more distinct discrimination among component sequences, which corresponds to superior decomposition quality. The specific calculation for *SSD* is given in Eq. (5).


$$\begin{array}{c} \begin{array}{c} SSD\;=\;{max}_{i<j\;}\left|\rho (s_{i},{\;s}_{j})\right|\; \end{array} \end{array}$$ (5)

where *s*_i_ and *s*_j_ are the *i*-th and *j*-th seasonal components obtained by seasonal-trend decomposition, respectively; *ρ*(*s*_*i*_, *s*_*j*_) denotes the Pearson correlation coefficient of the two seasonal components. The specific calculations for *Fuzzy_ Entropy_Sum* are given in Eqs. (6) and (7). Specifically, we first calculate the fuzzy entropy values corresponding to the trend term and each seasonal sub-term according to Eq. (6).


$$\begin{array}{c} FuzzyEn\left(m,\;r,\;n\right)\;=\;-𝐥𝐧(\frac{\phi_{m+1}(r,\;n)}{\phi_{m}(r,\;n)}) \end{array}$$ (6)

where ϕ_*m*_(*r*, *n*) represents the *m*-dimensional embedded average similarity; *r* is the similarity tolerance coefficient and *n* is the scale factor. Subsequently, the above-computed results are substituted into Eq. (7) to obtain the *Fuzzy_ Entropy_Sum metric* value of the decomposition algorithm.


$$\begin{array}{c} Fuzzy\_Entropy\_Sum\;=\;{FuzzyEn}_{Trend}\;+\;\sum_{k=1}^{K}{FuzzyEn}_{Season}^{k} \end{array}$$ (7)

where *Fuzzy_Entropy_Sum* is defined as the sum of the fuzzy entropies of the trend term and each seasonal term; *FuzzyEn*_*Trend*_ is the fuzzy entropy result of the trend term; ${FuzzyEn}_{Season}^{k}$ is the fuzzy entropy result of the *k*-th seasonal term.

The overall execution process of this decomposition algorithm is as follows:

Step 1: Perform power spectral density analysis on the PM2.5 concentration series in the training set by city unit to adaptively determine the period-factor parameters for MSTL decomposition.

Step 2: Define the search-optimization intervals of seasonal-smoothing-window parameters corresponding to different seasonal-period factors.

Step 3: Based on the search-optimization intervals determined in Step 2, take the *SSD* index as the objective optimization value, and adopt the grid-search optimization strategy to carry out GSPMSTL pre-decomposition experiments for the PM2.5 concentration series in the training set, so as to determine the optimal seasonal- smoothing-window parameters of this decomposition algorithm. Further validation of the obtained results is conducted using the *Fuzzy_Entropy_Sum* index.

Step 4: Take the initialized period-factor and seasonal-smoothing-window parameters obtained from Step 1 and Step 2 as common initialization parameters, and independently implement MSTL decomposition for the PM2.5 concentration series in the training subset, validation subset and test subset respectively to acquire the final decomposition results for each divided subset.

### FATA

The FATA is a novel metaheuristic optimization algorithm proposed in 2024 [42]. This algorithm optimizes problem parameters through two strategies: population search and individual search, which enhances the global search capability of the algorithm. The pseudocode execution process is shown in S2 Table.

In this study, we adopt the FATA to optimize seven hyperparameters of the CNN-LSTM hybrid model, including training batch size, rolling window size, optimizer type, number of Conv1D layers, number of LSTM layers, number of hidden LSTM units and learning rate. These hyperparameters exert critical regulatory effects on the model’s convergence speed and accuracy, as well as its capacity for extracting complex temporal features and fitting temporal memory patterns, and serve as core parameters that determine the prediction accuracy and performance stability of the CNN-LSTM hybrid model. Drawing on the hyperparameter setting experience in the field of PM2.5 time series prediction and taking into account that an excessively large hyperparameter search space will incur substantial time overhead, we ultimately define the specific optimization intervals for each hyperparameter. The initialization parameter settings of the FATA and the configuration of hyperparameter optimization intervals are presented in Table 2.

**Table 2:** Initialization parameter settings of the FATA.

Parameter type	value
Iteration	30
Population size	10
Fitness objective	*RMSE* of validation set
Refractive index	0.2
Target optimization parameters	Batch size: [8, 64]
	Rolling window size: {12, 24, 36, 48}
	Optimizer type: {Adam, Adagrad}
	Number of Conv1D layers: {1, 2}
	Number of LSTM layers: {1, 2}
	Number of hidden LSTM units: [32, 128]
	Learning rate: [0.001, 0.01]

### Convolutional neural network

Convolutional neural networks are feed-forward deep learning models that can automatically learn the spatial hierarchy and local features of input data. CNN screens effective features by performing sliding convolution on data with multiple convolution kernels, and has significant advantages in data feature extraction [43].

### Long short-term memory network

LSTM is a special recurrent neural network that introduces a gating mechanism to solve the gradient vanishing or exploding problems existing in RNN [44]. Its gating unit consists of three parts: input gate, forget gate and output gate. The input gate is used to control the update degree of the current input information to the cell state. The forget gate is used to control the forgetting amount of the cell state information at the previous moment. The output gate determines the output result of the information flow of the LSTM gating unit according to the current input and historical information.

### Model evaluation indicators

Four indicators are selected to evaluate the prediction accuracy of each model: Root Mean Square Error (*RMSE*), Correlation Coefficient (*R*^2^), Mean Absolute Error (*MAE*), and Mean Absolute Percentage Error (*MAPE*), as shown in Eqs. (8)-(11), where *n* is the number of samples, *y*_i_ is the actual value, and ${\hat{y}}_{i}$ is the predicted value.


$$\begin{array}{c} \begin{array}{c} MAPE\;=\;\frac{1}{n}\sum_{i=1}^{n}\left|\frac{{\hat{y}}_{i}\;-\;y_{i}}{y_{i}}\right| \end{array} \end{array}$$ (8)

$$\begin{array}{c} \begin{array}{c} R^{2}\;=\;1\;-\;\frac{\sum_{i=1}^{m}{({\hat{y}}_{i}\;-\;y_{i})}^{2}}{\sum_{i=1}^{m}{(y_{i}\;-\;{\overset{―}{y}}_{i})}^{2}} \end{array} \end{array}$$

$$\begin{array}{c} \begin{array}{c} RMSE\;=\sqrt[{\frac{1}{n}}]{\sum_{i=1}^{n}{({\hat{y}}_{i}\;-\;y_{i})}^{2}} \end{array} \end{array}$$ (10)

$$\begin{array}{c} \begin{array}{c} MAE\;=\;\frac{1}{n}\sum_{i=1}^{n}\left|{\hat{y}}_{i}\;-\;y_{i}\right| \end{array} \end{array}$$ (11)

### Overall workflow of the model

**Fig 3:**
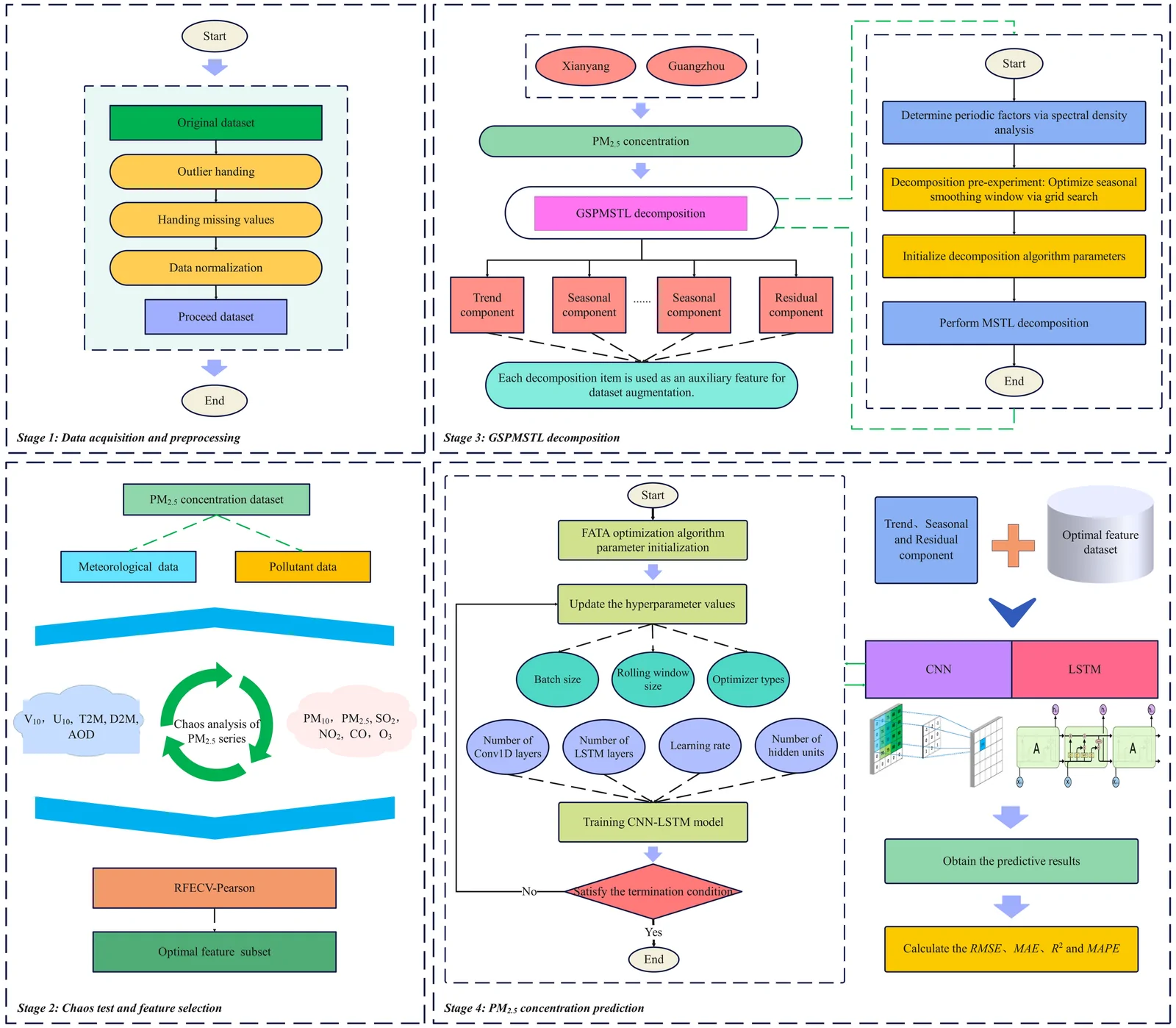
Framework of data processing for FATA-GSPMSTL-CNN-LSTM model.

As shown in Fig 3, the data processing flow of the proposed model is divided into four stages: First, preprocess the original dataset, mainly including outlier processing, missing value processing and normalization.Second, perform feature selection based on the RFECV-Pearson strategy to determine the optimal feature descriptor set for each city’s dataset. Third, adaptively decompose the PM2.5 concentration series into one trend term, several seasonal terms and one residual term using the GSPMSTL algorithm, and reconstruct the dataset by taking them as extended features. Fourth, taking each city as a unit, seven target hyperparameters of the CNN-LSTM hybrid model, including batch size, rolling window size, optimizer type, number of Conv1D layers, number of LSTM layers, number of LSTM hidden units, and learning rate, are iteratively optimized using the FATA, and the finally-trained model is saved. Fifth, the target model is invoked to make predictions on the test sets of the two cities respectively. Four performance evaluation metrics are calculated based on the prediction results so as to evaluate the model performance.

## Results

### RFECV-Pearson feature selection analysis

**Fig 4:**
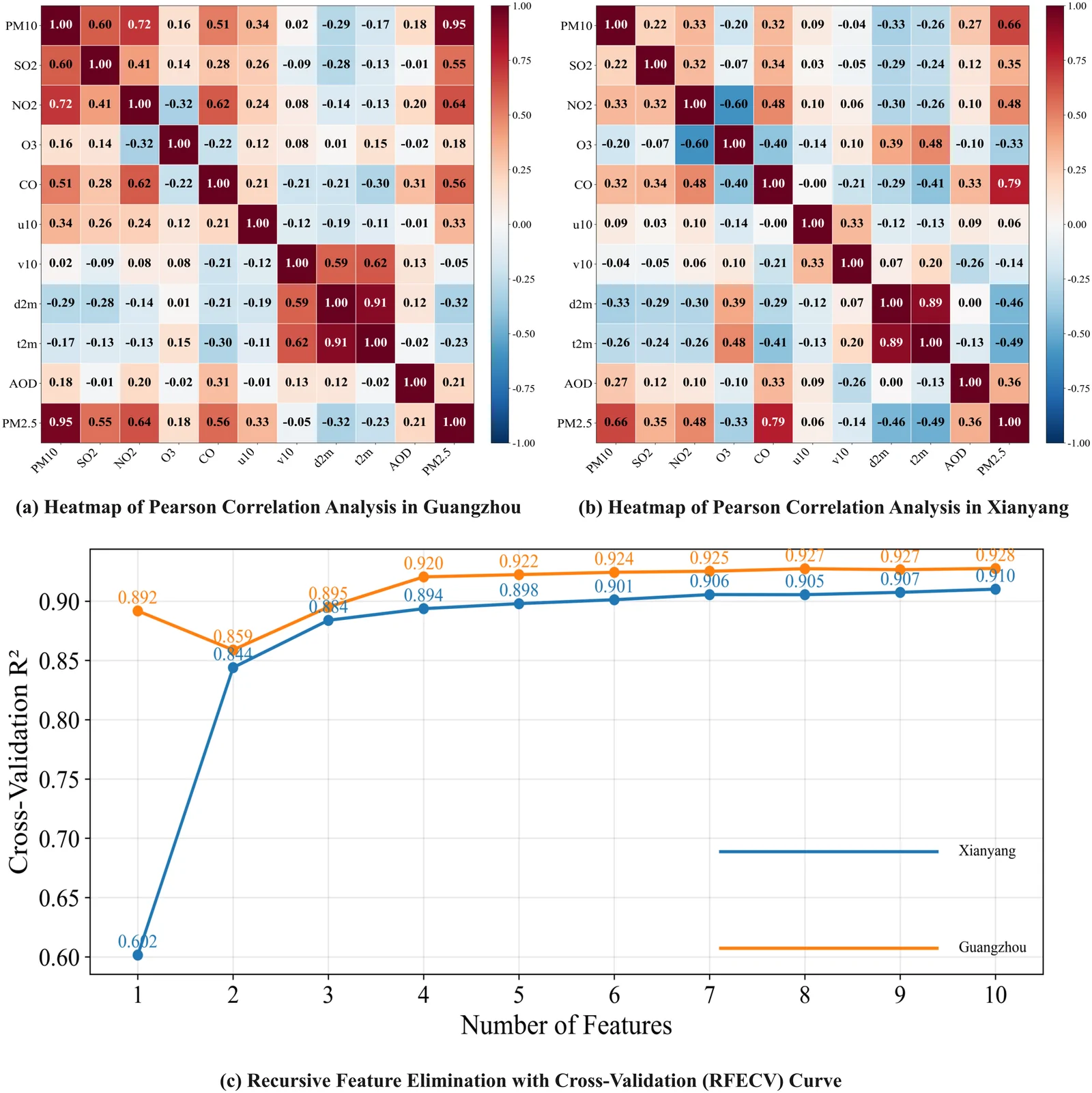
Results of RFECV-Pearson feature selection analysis.

To accurately select feature factors that contribute significantly to PM2.5 concentration prediction and thereby determine the PM2.5 feature descriptor set, the random forest model is used as the base model of the RFECV cross-validation method, combined with the Pearson correlation analysis method to improve the reliability of feature selection for the dataset. The results of RFECV cross-validation and Pearson analysis are shown in Fig 4.

It can be seen from Fig 4(a) and (b) that the PM2.5 concentration in Guangzhou is significantly positively correlated with four features: PM10, NO2, CO, and SO2, and strongly negatively correlated with dew point temperature (d2m) and air temperature (t2m). The PM2.5 concentration in Xianyang is significantly positively correlated with PM10, CO, and NO2, and strongly negatively correlated with dew point temperature and air temperature. Feature importance ranking was conducted based on the *R*^2^ index from RFECV, with the results shown in Table 3. The importance indices of PM10 are 0.9049 and 0.5262 under the prediction scenarios of Guangzhou and Xianyang respectively, contributing the most to the cross-validation results. CO ranks second with importance indices of 0.0242 and 0.3698 respectively. Features such as NO2, t2m, and AOD also contribute to a certain extent to the prediction accuracy of PM2.5. As shown in Fig 4(c), the *R*^2^ values of the cross-validation results tend to be stable when the number of features is 7 for both Guangzhou and Xianyang. Given the Pearson correlation coefficient of SO2 in Guangzhou reaches 0.55, SO2 is thus included in the feature descriptor set of Guangzhou as well. Therefore, the feature descriptor set for Guangzhou is determined as {PM10, CO, NO2, t2m, AOD, v10, d2m, SO2}, and the feature descriptor set for Xianyang is {PM10, CO, t2m, d2m, AOD, NO2, v10}.

**Table 3:** Feature importance ranking based on cross-validation.

Rank	Guangzhou		Xianyang	
	Feature	Importance	Feature	Importance
1	PM10	0.9049	PM10	0.5262
2	CO	0.0242	CO	0.3698
3	NO2	0.0153	t2m	0.0415
4	t2m	0.0133	d2m	0.0175
5	AOD	0.0092	AOD	0.0099
6	V10	0.0083	NO2	0.0083
7	d2m	0.0079	v10	0.0081
8	O3	0.0079	O3	0.0066
9	u10	0.0056	u10	0.0062
10	SO2	0.0034	SO2	0.0060

### Chaotic characteristic analysis

**Fig 5:**
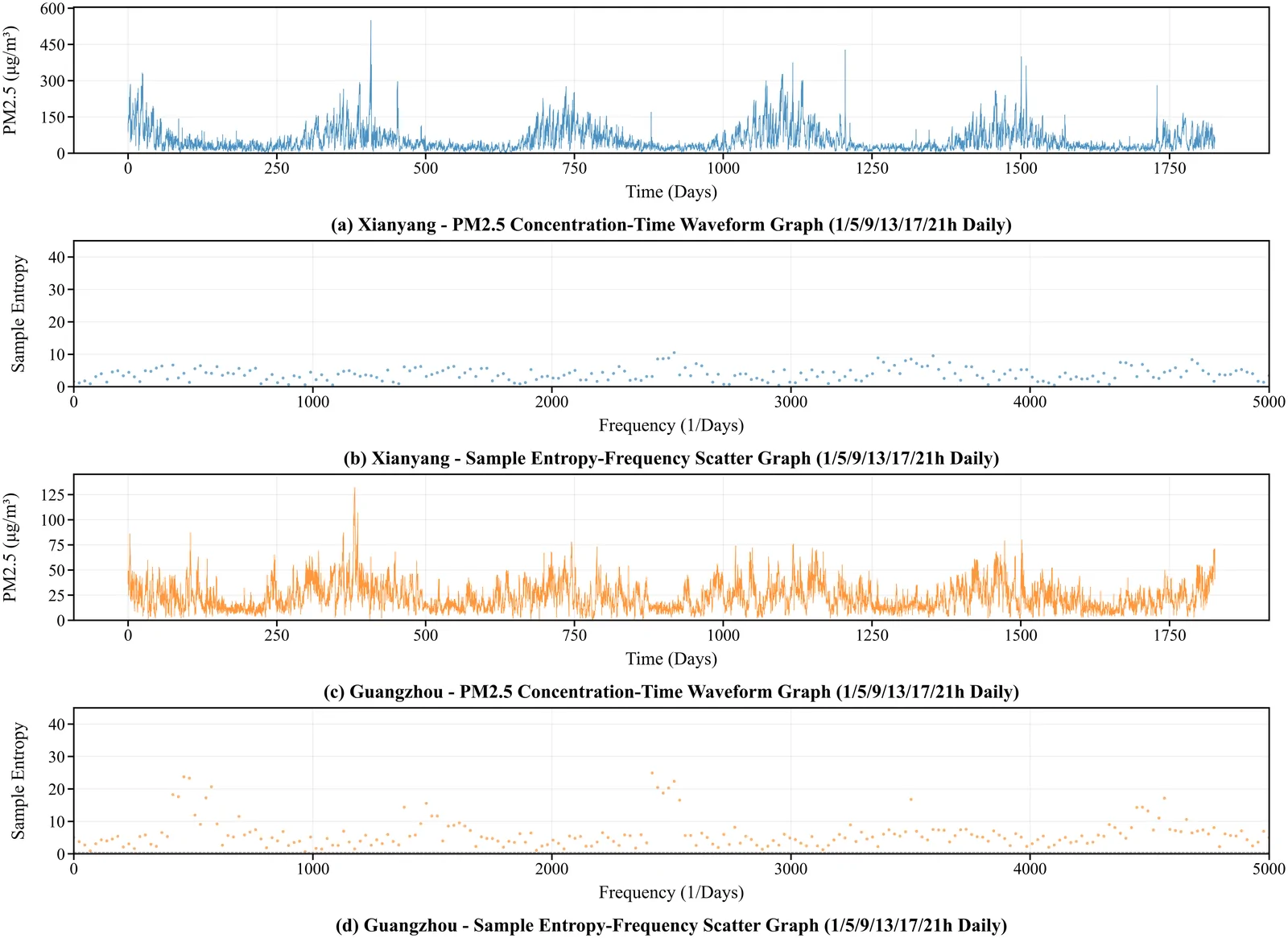
Time-domain and frequency-domain characteristics of hourly PM2.5 concentration in Xianyang and Guangzhou.

To effectively assess whether the PM2.5 concentration time series exhibits chaotic characteristics, chaotic characteristic analysis of PM2.5 was carried out in Xianyang and Guangzhou. The time-domain and frequency-domain diagrams of the two cities are shown in Fig 5.

Fig 5(a) and (c) show the temporal fluctuations of PM2.5 concentrations in Xianyang and Guangzhou. The occurrence times and amplitudes of the peaks and valleys in the PM2.5 concentration curves have no fixed regularity, and the concentration values are within a limited interval. This indicates that the PM2.5 concentrations in Xianyang and Guangzhou show aperiodic and non-random variation characteristics, and it can be preliminarily determined that they have chaotic characteristics.In addition, there are discrepancies in the fluctuation amplitudes of PM2.5 concentrations between the two cities at the same moment, which indicates that the evolution process of PM2.5 pollution exhibits spatiotemporal heterogeneity under prediction scenarios of different regions.It can be seen from Fig 5(b) and (d) that the frequency-sample entropy points corresponding to Xianyang and Guangzhou are concentrated in the limited ranges of 0–10 and 0–25, respectively, and the sample entropy values of all points are finite and non-zero. This demonstrates that the variation of the PM2.5 concentration series in the two cities is neither consistent with pure random noise nor with strictly periodic series. Kolmogorov entropy is an important numerical index for judging whether a time series has chaotic characteristics. If a time series is chaotic, its Kolmogorov entropy should be a non-zero finite value; otherwise, the series tends to be random. The calculated Kolmogorov entropy values of the PM2.5 concentration series in Xianyang and Guangzhou are 0.0232 and 0.0146, respectively, further confirming that the PM2.5 concentration series of both cities possess chaotic characteristics.

The results reveal that the PM2.5 concentration time series of both Guangzhou and Xianyang exhibit chaotic dynamic characteristics, and there exists obvious spatiotemporal heterogeneity in the PM2.5 pollution evolution patterns between the two cities. Chaotic PM2.5 time series generally contain complex multi-period nonlinear variation features. Direct modeling with a single model fails to fully extract their inherent intricate dynamic laws. In addition, the spatiotemporal heterogeneity of PM2.5 concentrations across different prediction scenarios imposes higher requirements on the model’s adaptability. The above analysis fully demonstrates that introducing an adaptive decomposition algorithm to assist time series modeling is theoretically reasonable.

### MSTL multiple seasonal-trend decomposition

#### Determination of seasonal periodic factors.

**Fig 6:**
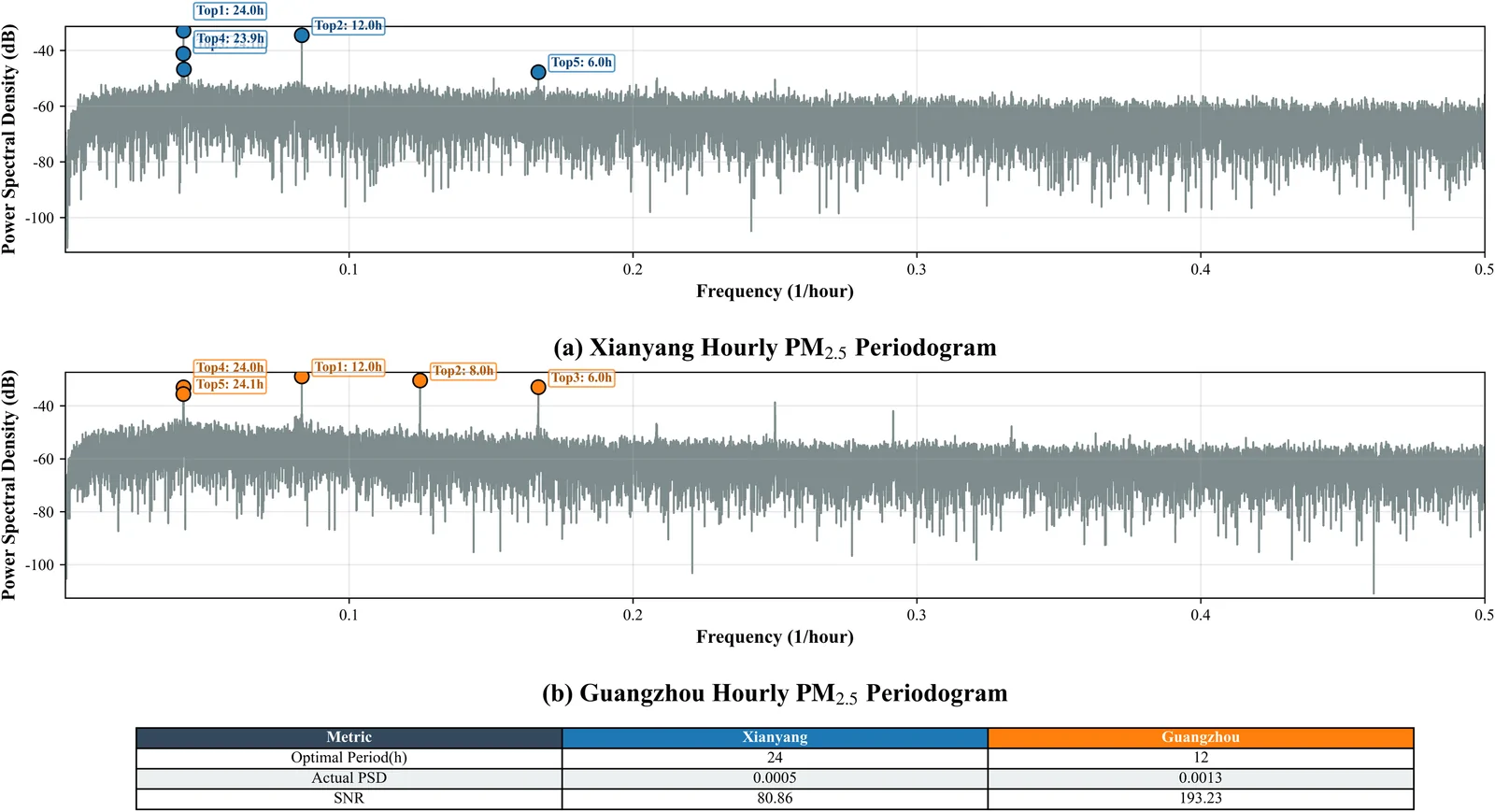
Periodograms of hourly PM2.5 concentration in Guangzhou and Xianyang.

Statistical analysis was performed on the PM2.5 concentration data of the two cities based on the periodogram method. The top 5 dominant periods sorted by energy spectral density were selected and displayed for each city, with the results shown in Fig 6.

It can be seen from Fig 6 that the integer dominant periods of the PM2.5 concentration time series in Xianyang, sorted by energy spectral density, are 24h, 12h, and 6h in sequence. For Guangzhou, the dominant periods are 12h, 8h, 6h, and 24h. This indicates that the variation pattern of PM2.5 concentration is dominated by the diurnal period (24h) in Xianyang and the semi-diurnal period (12h) in Guangzhou. Meanwhile, the *PSD* and *SNR* of Guangzhou are higher than those of Xianyang, suggesting that the periodic fluctuation of PM2.5 concentration in Guangzhou is more significant. In this study, for each city, considering that the power spectral density corresponding to 6h in Xianyang is relatively weak, only 24h and 12h are selected as the dominant periodic factors for the multi-seasonal decomposition algorithm of PM2.5 in Xianyang. Meanwhile, 12h, 8h, 6h, and 24h are chosen as the corresponding dominant periodic factors for Guangzhou.

#### GSPMSTL decomposition experiment.

Since it has been verified that the PM2.5 concentration series exhibits chaotic characteristics, based on the optimal seasonal factor combination, the grid search algorithm was adopted to carry out pre-decomposition experiments on the PM2.5 concentration series by city unit so as to determine the optimal seasonal smoothing window combination of the decomposition algorithm.

**Fig 7:**
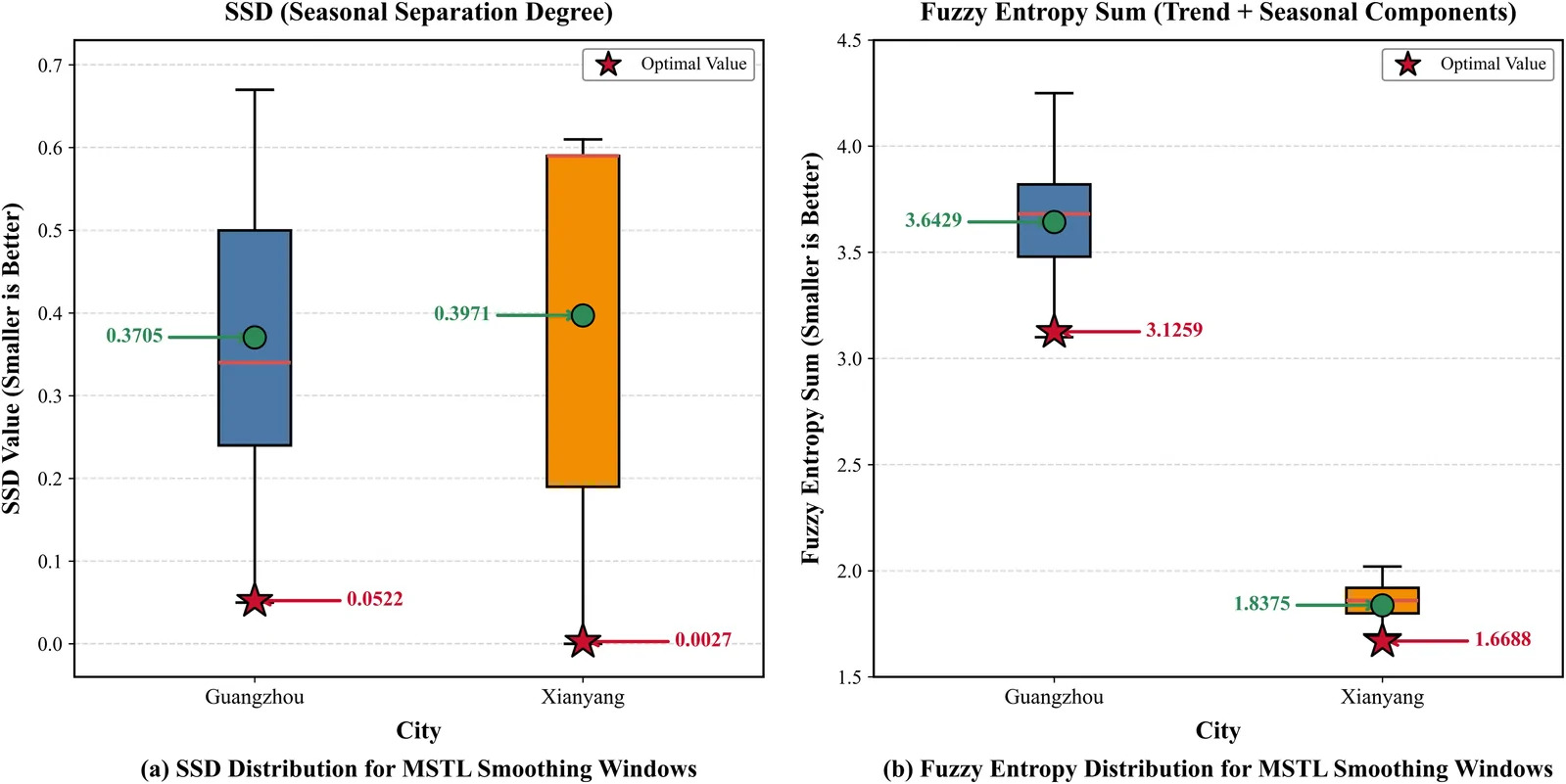
Distribution of decomposition quantitative indicators under different seasonal smoothing window combinations.

Combined with relevant literature on MSTL seasonal smoothing window settings and actual research requirements, the search spaces corresponding to seasonal periodic factors 12 and 24 in Xianyang were determined as {7, 9, 11, 13, 15, 17} and {15, 17, 19, 21, 23}, respectively. For Guangzhou, the search spaces corresponding to seasonal periodic factors 6, 8, 12, and 24 were {5, 7, 9, 11}, {7, 9, 11, 13, 15, 17}, {7, 9, 11, 13, 15, 17}, and {15, 17, 19, 21, 23}, respectively. The *SSD* was selected as the optimization index of the algorithm, and pre-experiments of MSTL decomposition were conducted on the training set of each city via the grid search algorithm, with the pre-decomposition results further examined based on the *Fuzzy_Entropy_Sum* index, to determine the optimal seasonal smoothing window parameters. The distribution of the *SSD* and *Fuzzy_Entropy_Sum* quantitative index for the decomposition results corresponding to all seasonal smoothing window combinations is shown in Fig 7.

It can be observed from Fig 7(a) that even under the same periodic factor parameters for the PM2.5 series in the same city, the decomposition quality varies greatly with different seasonal smoothing window combinations. The seasonal smoothing windows determined by the grid search method can ensure that the *SSD* decomposition evaluation index reaches the optimum. Meanwhile, to avoid the limitation of single-index evaluation, the *Fuzzy_Entropy_Sum* value of each combination was further calculated. Fig 7(b) shows the distribution of the *Fuzzy_ Entropy_Sum* index. The *Fuzzy_Entropy_Sum* values of the optimal smoothing window combinations in the two cities are 3.1259 and 1.6688, respectively, which are close to the optimal value of this index. Therefore, {5, 9, 11, 19} and {7, 23} were selected as the optimal seasonal smoothing window combinations for Guangzhou and Xianyang, respectively.

**Fig 8:**
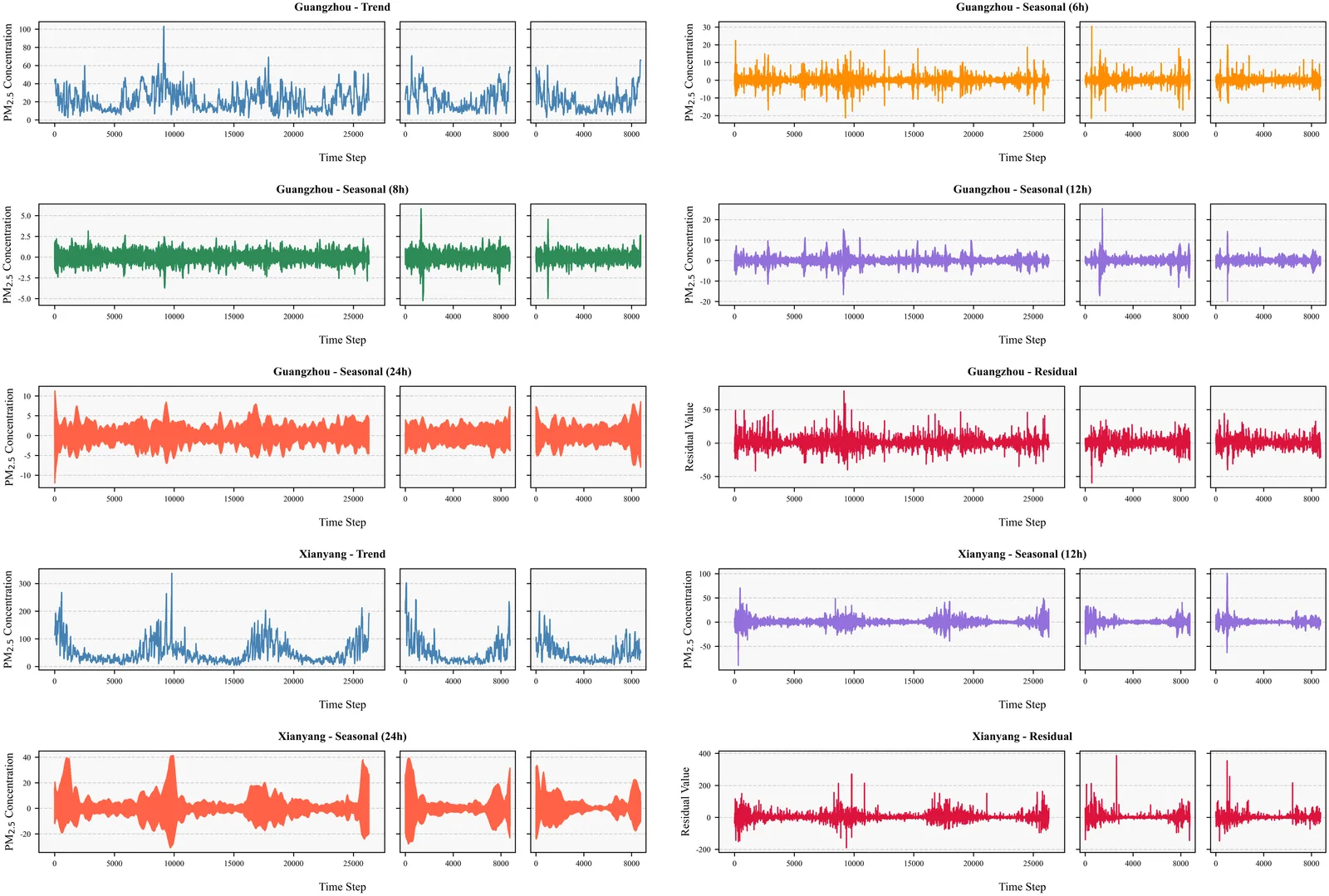
MSTL decomposition results of Guangzhou and Xianyang.

The parameter settings for MSTL decomposition are listed in S3 Table, where iterate, *p*, *n*_s,_ *n*_t_, and degree correspond to: the outer iteration number of the MSTL decomposition algorithm, seasonal periodic factor, seasonal smoothing window, trend smoothing window, and polynomial degree for smoothing fitting, respectively. With the decomposition initialization parameters determined in the above sections, we perform independent MSTL decomposition on the training, validation and test subsets of PM2.5 time series for Guangzhou and Xianyang on a city-by-city basis.The decomposition results are shown in Fig 8. The PM2.5 concentration time series corresponding to the training, validation and test subsets of both cities are independently decomposed into one trend term, multiple seasonal terms and one residual term. Meanwhile, the seasonal components at different periodic scales are well separated, which greatly improves the overall decomposition quality. Based on the above decomposition results, we performed feature reconstruction on the dataset by city-unit, and applied it to the subsequent modeling analysis.

### Sensitivity analysis of prediction results to hyperparameter optimization algorithms

To analyze the effect of optimization algorithms on model performance and further identify the optimal hyperparameter optimization strategy for the CNN-LSTM model, we replaced the FATA in the M8 model by introducing the WOA and SSA, and constructed the WOA-GSPMSTL-M5 and SSA-GSPMSTL-M5 combined models, respectively. First, by referring to the hyperparameter-setting experience reported in existing literature and taking the time-space cost overhead during the model training phase into account, we finally determined the optimal values of non-optimizable hyperparameters for the CNN-LSTM model, with relevant configuration information listed in Table 4.

**Table 4:** Non-optimized hyperparameters settings of the target model.

Parameter types	Value	Parameter types	Value
Random seed	42	Filters	64
Epoch	50	Kernel size	3
Early stop patience	10	Activation function	ReLU
Normalization	MinMaxScaler	Dropout	0.2
Data split ratio	Train: Validation: Test = 3: 1: 1	Process type	AMD Ryzen 7 8745H (3.80 GHz)
Loss function	*MSE*	Python interpreter	Python 3.12
Pooling type	Max pooling	Core library versions	Tensorflow 2.20.0
Pool size	2		Keras 3.13.2

**Fig 9:**
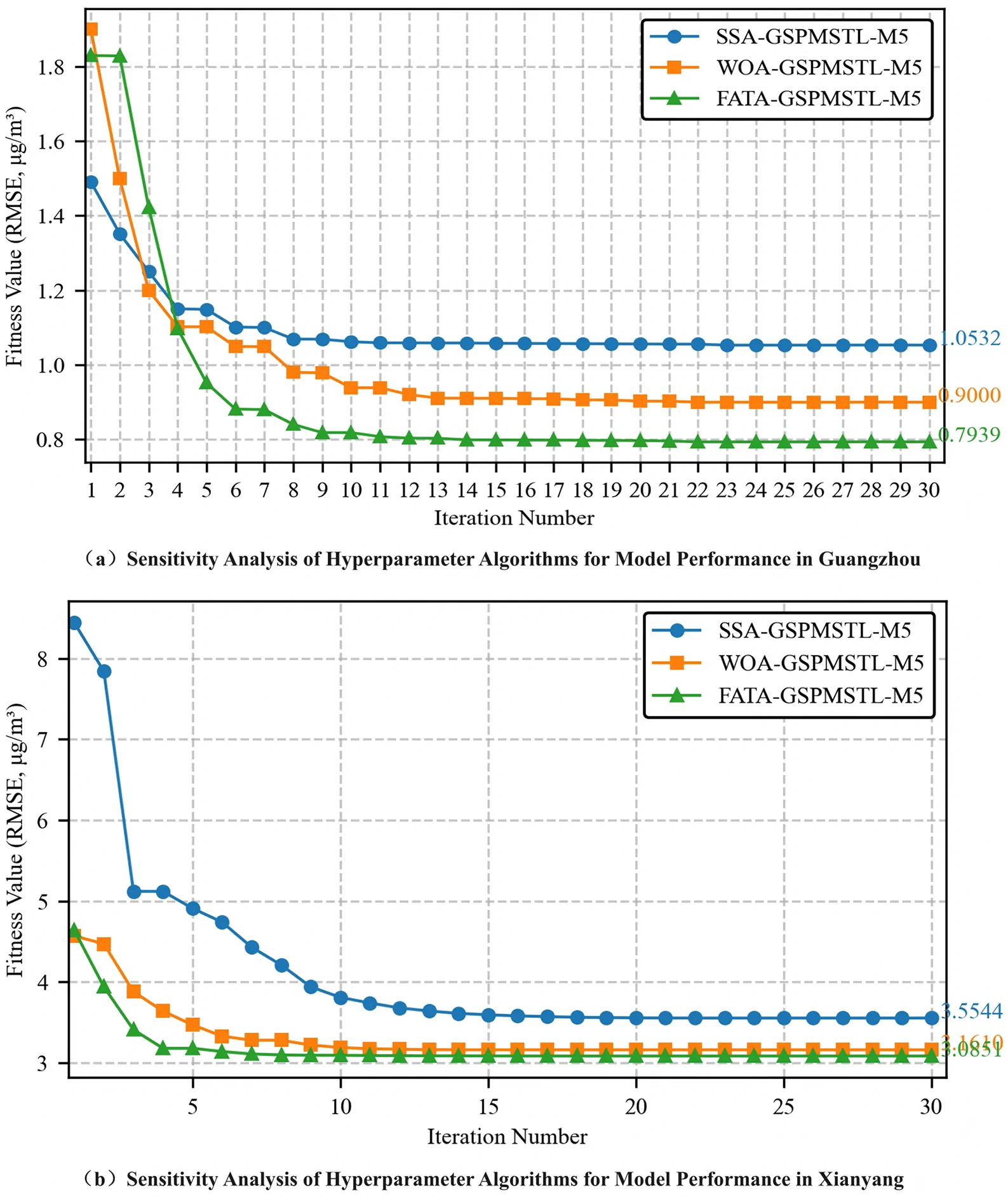
Comparison of convergence results for different heuristic algorithms.

Subsequently, we independently repeated the sensitivity-analysis experiments of each combined model five times for different prediction scenarios to effectively avoid the interference of random factors on model prediction results (The convergence results of each model from the five repeated experiments are shown in S1-S5 Figs, respectively), and selected the run with the optimal convergence accuracy of the M8 model for display (see Fig 9). Fig 9 presents the *RMSE* fitness convergence curves of each model on the validation set. As shown in Fig 9(a), in the sensitivity-analysis experiment for Guangzhou, the three models tended to converge after the 13th iteration. Among them, the FATA-GSPMSTL-M5 combined model yielded an *RMSE* value of 0.7939 at the end of the 30th iteration, which decreased by 24.62% and 11.79% compared with the models corresponding to the SSA and WOA, respectively. In the Xianyang experiment shown in Fig 9(b), the combined models optimized by WOA and FATA tended to converge after the 10th iteration, and exhibited overall faster convergence speed than the SSA-GSPMSTL-M5 model. In addition, at the end of the 30th iteration, the *RMSE* value obtained by FATA decreased by 13.20% and 2.40% relative to the SSA-GSPMSTL-M5 and WOA- GSPMSTL-M5 combined models, respectively. The above results indicate that the type of hyperparameter optimization algorithm exerts an influence on model prediction accuracy. Compared with the SSA and WOA, FATA delivers relatively superior convergence accuracy and convergence speed. This may be attributed to the following reasons: FATA can automatically filter inferior individuals according to the definite-integral population quality factor to screen population quality, retain more high-quality individuals during iterations, and avoid invalid searches, thereby achieving a high convergence speed. Meanwhile, the total-internal-reflection strategy can effectively prevent the population from falling into local optima, which further ensures the relative advantage of the FATA in terms of convergence accuracy.

Subsequently, we selected the models with the optimal convergence-accuracy performance from independent repeated experiments for the SSA, WOA and FATA, and further examined the sensitivity-analysis results and screened the optimal hyperparameter optimization strategy on the test set, as shown in Table 5.

**Table 5:** Prediction performance of various heuristic optimization algorithms.

Model	Guangzhou				Xianyang			
	*RMSE*	*R* ^2^	*MAE*	*MAPE*	*RMSE*	*R* ^2^	*MAE*	*MAPE*
SSA-GSPMSTL-M5	1.4528	0.9778	1.1583	9.1845	3.8247	0.9843	2.6031	7.4826
WOA-GSPMSTL-M5	1.3812	0.9812	1.0985	8.6976	3.6154	0.9851	2.4689	7.0851
FATA-GSPMSTL-M5	1.2687	0.9845	1.0071	7.9986	3.5285	0.9869	2.4013	6.9205

The analysis reveals that the FATA-GSPMSTL-M5 model exhibits relatively optimal prediction performance: compared with the SSA-GSPMSTL-M5 and WOA-GSPMSTL-M5 models, its *MAPE* errors decreased by 12.91% and 8.04% for Guangzhou, and by 7.51% and 2.32% for Xianyang, respectively. The above results fully verify that hyperparameter optimization using the FATA can effectively improve model prediction performance compared with other optimization strategies. Accordingly, we take FATA-GSPMSTL-M5 as the target model (namely Model M8) for further comparative analysis and the optimal hyperparameter combination obtained by FATA is listed in Table 6.

**Table 6:** Optimal hyperparameter combination of the target model.

City	Target optimization parameters	Optimization results
Guangzhou	Batch size	16
	Rolling window size	48
	Optimizer type	Adam
	Number of Conv1D layers	1
	Number of LSTM layers	1
	Number of hidden LSTM units	64
	Learning rate	0.002
Xianyang	Batch size	32
	Rolling window size	48
	Optimizer type	Adam
	Number of Conv1D layers	1
	Number of LSTM layers	1
	Number of hidden LSTM units	32
	Learning rate	0.001

### Comparative evaluation of cross-model results

Before carrying out cross-model comparative analysis, to fully guarantee the fairness of prediction-performance comparison among different models, we first identified the types of hyperparameters to be optimized for baseline models and their corresponding optimization ranges. Subsequently, the grid-search optimization algorithm was adopted to determine their respective optimal hyperparameter combinations, with relevant information listed in S4 Table. Finally, the optimized optimal baseline models together with the target model were subjected to cross-model comparative analysis.

As can be seen from S6 Fig, the fitting scatter points of models M1–M5 are relatively scattered in the regression plots of the two cities. The prediction performance of the combined model M5 is superior to that of the single models, but there is still room for improvement. After being optimized by introducing the decomposition algorithm, the fitting performance of models M6–M8 is significantly improved. Among them, the fitting scatter points of model M8 are the most concentrated and approximately distributed along the 45° diagonal line, demonstrating its optimal prediction performance.

**Fig 10:**
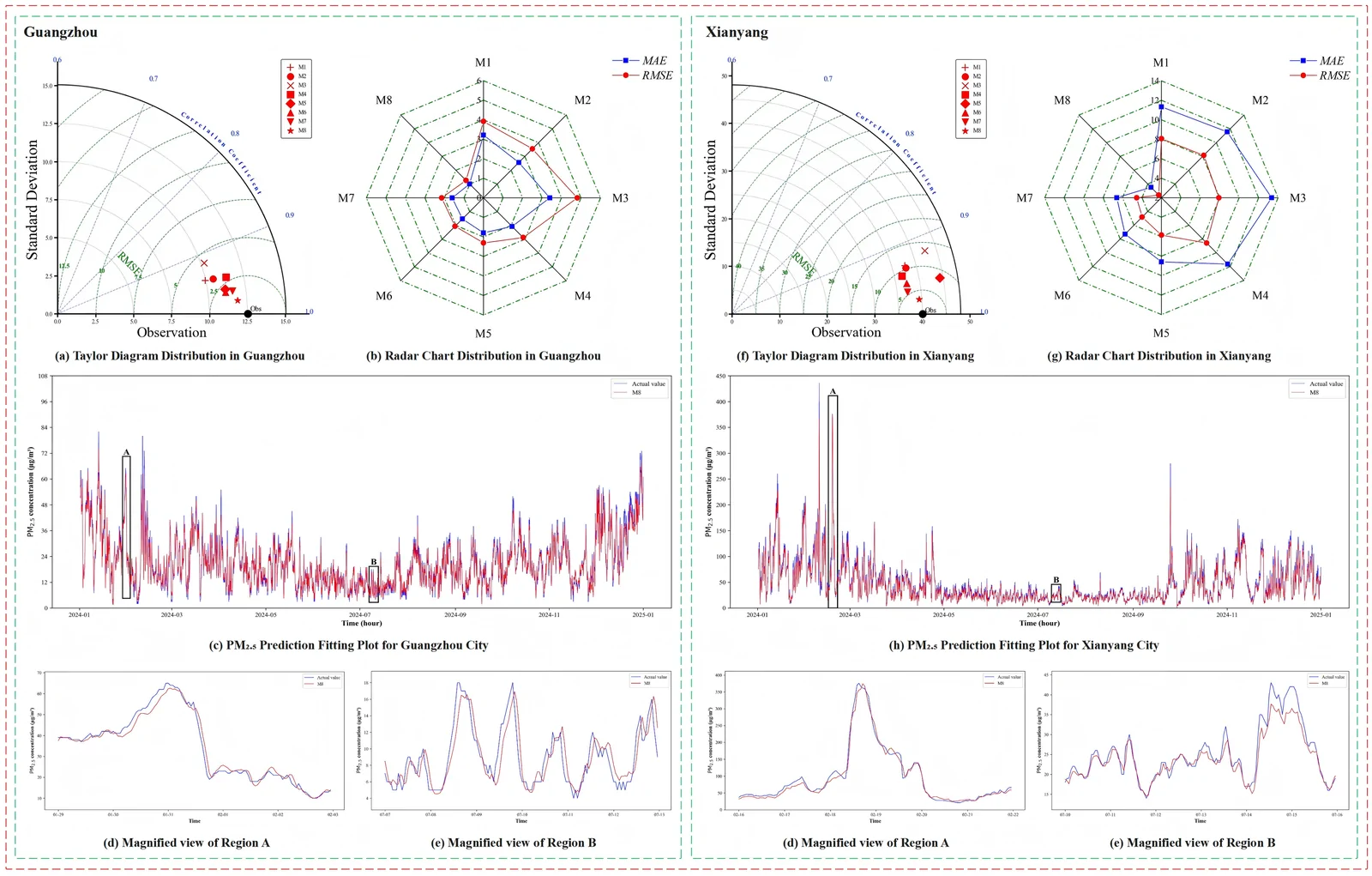
Multi-scale prediction performance comparison across models.

As shown in Fig 10(a), the *RMSE* errors of models M5-M8 on the test set of Guangzhou are all less than 2.5 μg/m³, which are overall superior to those of models M1-M4. Among them, model M8 is the closest to the observed values (Obs), indicating that M8 yields a smaller *RMSE* error compared with other models, and its predicted-value series is also more consistent with the observed-value series in terms of fluctuation characteristics. As can be seen from Fig 10(f), under the prediction scenario of Xianyang, only model M8 achieves an *RMSE* below 5 μg/m³; meanwhile, its Pearson correlation coefficient reaches as high as 0.9970, demonstrating a relatively higher linear correlation between its predicted series and observed series. It can be observed from Fig 10(b) and (g) that the closed curves in the error radar charts for Guangzhou and Xianyang both exhibit obvious depressions in the directions of M6-M8, reflecting that these models obtain relatively lower *MAE* and *MAPE* error values than baseline models M1-M5. This indicates that applying modal decomposition to the PM2.5 time-series-modeling task can effectively improve the prediction performance of the model. Among them, the closed curve presents the deepest-level depression in the direction of model M8 relative to models M7 and M6, which suggests that GSPMSTL contributes a more prominent improvement to the prediction performance of the CNN-LSTM combined model compared with the two modal decomposition strategies of EMD and VMD. Fig 10(e) and (h) illustrate the PM2.5-concentration prediction-fitting performance of model M8 under the prediction scenarios of Guangzhou and Xianyang, respectively. There exists evident spatiotemporal heterogeneity in the PM2.5-concentration- variation characteristics of the two cities: the annual-average PM2.5 concentration in Xianyang is significantly higher than that in Guangzhou, and its overall fluctuation amplitude is more intense. By analyzing the fitting performance of model M8 over high and low-PM2.5-concentration segments, it can be found that model M8 can accurately fit the peaks, valleys and smooth-variation segments of the observed PM2.5 concentration curves, which effectively verifies that the target model M8 possesses excellent fitting performance under diverse prediction scenarios.

To comprehensively evaluate the prediction performance of the models under different working conditions, we further divided the test set into two periods: heating season (January, February, March, November, December) and non-heating season (April–October). The prediction performance of the models in different periods was evaluated based on the four evaluation indicators, with the results shown in Table 7. In the prediction scenario of Xianyang, M4 and M5 show improved prediction accuracy compared with M1–M3. In the heating season, M5 outperforms M4 by 30.24%, 6.22% and 45.63% in *RMSE*, *R*^2^ and *MAE*, respectively. In the non-heating season, the improvements are 16.26%, 4.62% and 4.28%, indicating that CNN can enhance the ability of LSTM to extract temporal features. Models M6–M8 using the decomposition algorithm achieve significant performance improvements, among which M8 performs the best. Compared with the suboptimal model M7, M8 achieves improvements of 42.45%, 2.43%, 44.17%, 36.07% in the heating season and 44.13%, 3.04%, 42.76%, 30.76% in the non-heating season, respectively. In the heating-season prediction scenario of Guangzhou, compared with the base model M5 and the suboptimal model M7, the target model M8 achieves improvements of 1.81% and 37.77%, as well as 1.89% and 38.59%, in terms of *R*^2^ and *MAE* indicators, respectively. In the non-heating-season prediction scenario, the corresponding improvements reach 5.33% and 44.61%, as well as 2.24% and 24.73%.

**Table 7:** Evaluation metrics of prediction models for two cities.

Season type	City	Model type	Performance Metrics			
			*RMSE*	*R* ^2^	*MAE*	*MAPE*
Heating season	Guangzhou	M1	4.4157	0.9079	3.5578	21.3874
		M2	4.4629	0.9059	3.3496	14.7878
		M3	6.4646	0.8026	4.8874	19.2161
		M4	3.5148	0.9416	2.5380	12.4367
		M5	2.4504	0.9716	1.8002	9.6738
		M6	2.6120	0.9678	1.9961	7.4739
		M7	2.4157	0.9711	1.8242	8.1912
		M8	1.4813	0.9895	1.1203	5.7527
	Xianyang	M1	15.3817	0.8875	12.3441	23.4491
		M2	14.6926	0.8974	11.3460	17.1428
		M3	17.7101	0.8509	10.5942	19.0205
		M4	15.0148	0.8928	11.4772	18.9990
		M5	10.4741	0.9479	6.2404	10.5545
		M6	8.9996	0.9615	5.7565	9.6292
		M7	8.6059	0.9648	6.5388	9.9438
		M8	4.9531	0.9882	3.6507	6.3571
Non-heating season	Guangzhou	M1	3.5089	0.8240	2.9725	28.6318
		M2	2.7241	0.8989	2.0041	13.6219
		M3	3.1486	0.8583	2.3501	16.3538
		M4	2.3257	0.9227	1.7414	12.4359
		M5	2.2029	0.9306	1.7899	16.0583
		M6	1.7349	0.9570	1.3165	8.5972
		M7	1.6989	0.9587	1.3172	10.9310
		M8	1.1518	0.9802	0.9914	9.2706
	Xianyang	M1	7.1974	0.8991	5.0217	21.2095
		M2	8.8683	0.8469	6.6176	22.0294
		M3	9.0583	0.8402	6.0244	24.9308
		M4	8.3052	0.8657	5.8070	21.5704
		M5	6.9502	0.9059	5.5582	25.4829
		M6	5.7549	0.9355	4.1460	18.7953
		M7	4.6897	0.9572	3.1330	10.5348
		M8	2.6201	0.9863	1.7933	7.2944

The above results indicate that model M8 possesses certain advantages in prediction accuracy and generalization performance under diverse prediction scenarios and application working-conditions compared with other baseline models, which confirms that the FATA and GSPMSTL modal decomposition can effectively improve the prediction performance of the model under complex application scenarios.

### Statistical significance test

In this study, the Diebold-Mariano (DM) statistical significance test was carried out for all models from three dimensions including the overall test set, heating season and non-heating season, so as to ensure the reliability of the cross-model comparative experimental results. Specifically, mean squared error (*MSE*) was selected as the loss function for the DM test, and the prediction step was set to 1. Meanwhile, the Newey-West robust variance estimation method was adopted to eliminate the impact caused by serial autocorrelation, and the two-sided normal Z-test was used to systematically evaluate the statistical significance of model prediction results. The DM statistical test results of the models under the overall test set, heating season and non-heating-season periods are presented in Table 8, S5 and S6 Tables, respectively.

**Table 8:** Results of model significance statistical test in the entire period.

Target model	Comparison model	Guangzhou		Xianyang	
		DM value	P value	DM value	P value
M8	M1	29.6630	0.0000	20.0281	0.0000
	M2	20.0042	0.0000	7.3889	0.0000
	M3	35.5714	0.0000	24.2491	0.0000
	M4	28.9732	0.0000	19.4057	0.0000
	M5	18.9210	0.0000	12.5747	0.0000
	M6	24.2563	0.0000	17.9532	0.0000
	M7	21.0887	0.0000	3.8976	0.0001

According to the table analysis, in the two cities of Guangzhou and Xianyang, the Diebold-Mariano (DM) statistics of model M8 compared with all other baseline models are positive. Among them, the DM statistical results of model M8 versus M7 under the entire-period test set are 21.0887 and 3.8976, respectively, while the DM statistical results compared with other baseline models are generally higher than 15. This indicates that M8 is overall superior to other baseline models in prediction performance, and this performance advantage is particularly significant for models M1-M6. In addition, the p-value of model M8 against M7 in the non-heating-season prediction scenario of Xianyang City is 0.0005, and the p-values of the remaining models under different dimensions all do not exceed this value, which is far lower than the significance threshold of 0.05. The above results show that at the 0.05 significance level, the null hypothesis that there is no significant difference in prediction accuracy between the target model and the baseline models can be rejected. This result effectively rules out the possibility that the performance improvement of the target model arises from random chance, and statistically verifies the reliability of the conclusion that “-the target model M8 is superior to all baseline models in terms of prediction performance”-.

### Model prediction ability under different prediction steps

**Fig 11:**
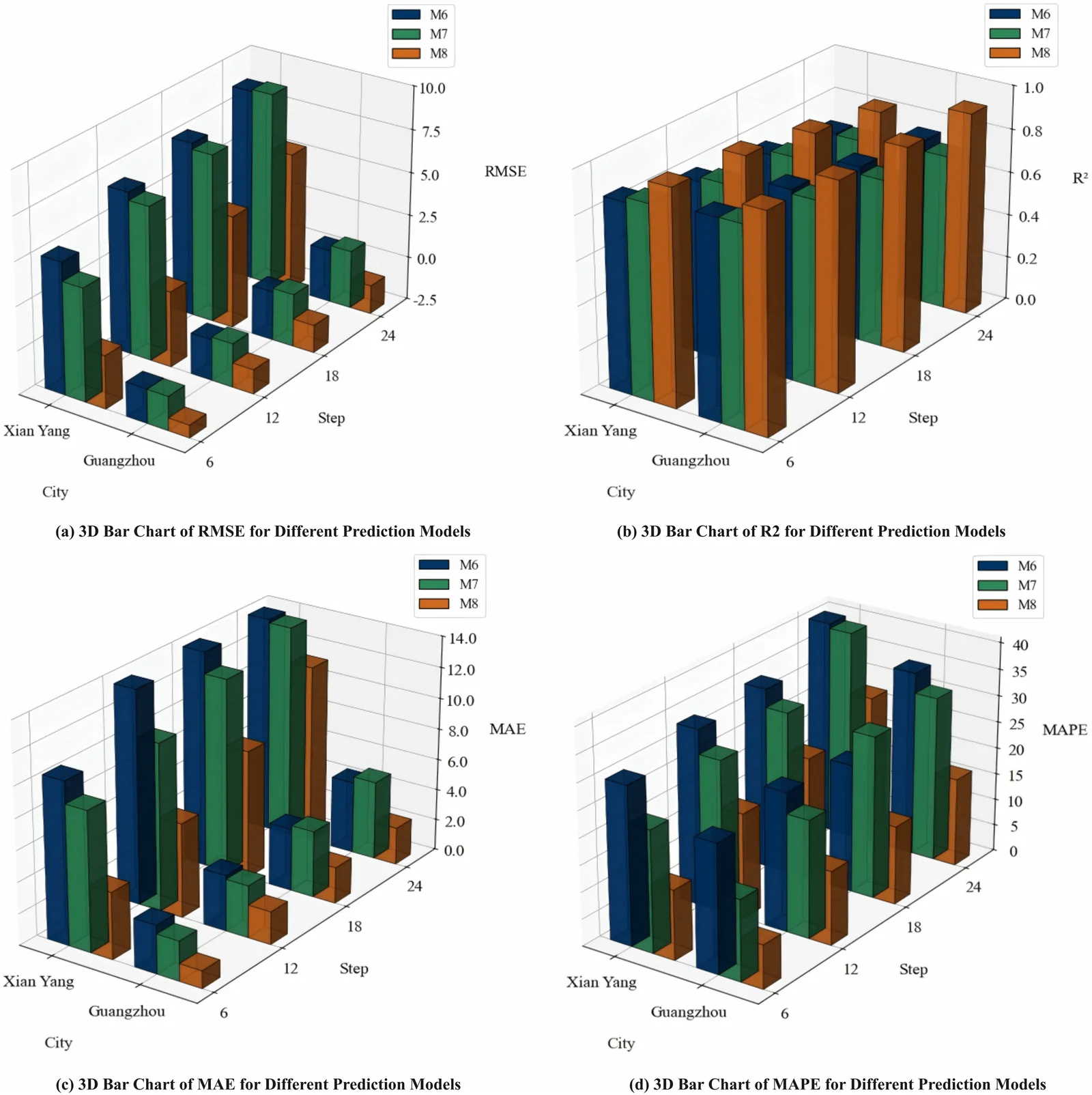
Performance comparison results of prediction steps for different models.

To further verify the prediction performance of the proposed model, this study selected models M6–M8 with the best prediction performance. Based on the datasets of Guangzhou and Xianyang, four prediction steps (6, 12, 18, and 24 hours) were adopted to train corresponding adaptive models. Their performance under different prediction steps was compared and evaluated on the whole test set, with the results shown in Fig 11.

Fig 11 presents the variation of prediction indicators of M6–M8 with prediction steps. The overall prediction performance of the models gradually degrades as the prediction step increases. Among them, the performance degradation rate in Xianyang is faster than that in Guangzhou, which is closely related to the higher prediction uncertainty caused by the higher average PM2.5 concentration and larger fluctuation amplitude in Xianyang. Meanwhile, M8 achieves higher prediction accuracy and a slower performance degradation rate than M6 and M7 for PM2.5 concentration prediction in both cities, indicating that the target model M8 possesses relative advantages in terms of performance stability and generalization ability.

### Ablation experiment

**Fig 12:**
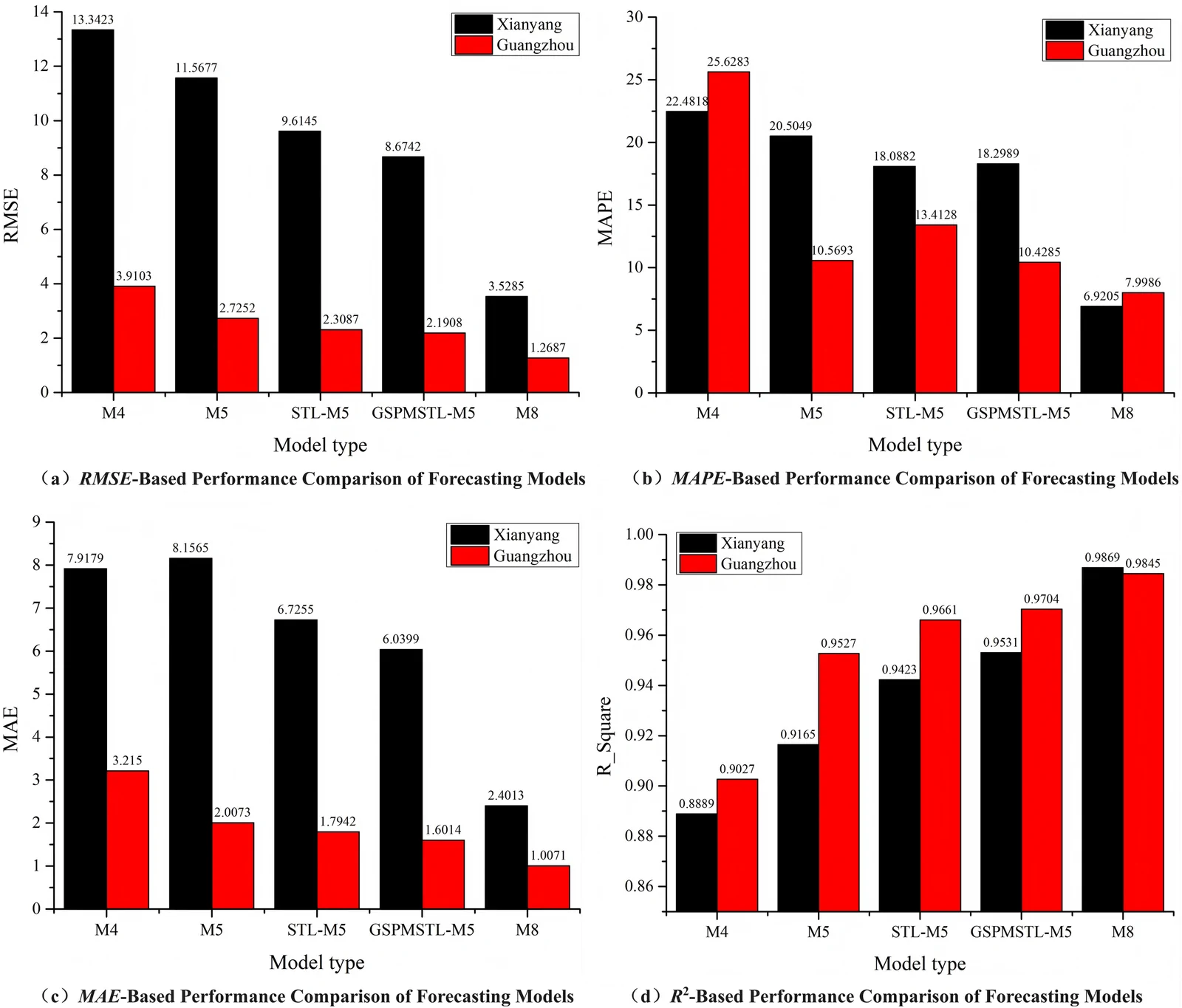
Comparison results of model ablation experiments.

To fully verify the effectiveness of each module within the target model M8 for improving model performance, this study conducts ablation experiments on model M8. Specifically, by removing the FATA module inside model M8 and adopting the single-seasonal-trend decomposition strategy (STL) to replace the GSPMSTL decomposition strategy, two simplified variants are constructed respectively: GSPMSTL-M5 and STL-M5 (the periodic factor parameter of the STL decomposition algorithm is empirically set to 24, and other initialization parameters are determined based on the default mapping mechanism built into the algorithm). These two simplified variants, together with models M4, M5 and M8, are subjected to comparative performance analysis for the entire period of the test set, and the results are shown in Fig 12.

It can be analyzed that model M8 achieves a significant improvement in prediction performance compared with the simplified variants GSPMSTL-M5 and STL-M5. Among them, their *R*^2^ prediction accuracy decreases by 1.43%, 3.43% and 1.87%, 4.52% respectively in Guangzhou and Xianyang relative to model M8, while the *MAPE* error indicators increase by 67.69%, 161.19% and 30.38%, 164.42%, respectively. The above results indicate that the multi-seasonal-trend decomposition strategy possesses more advantages in assisting the model to boost prediction performance compared with the single-seasonal-trend decomposition strategy. Enhancing the adaptive capability of the decomposition algorithm based on power spectral density analysis and meta-heuristic search optimization strategy can also effectively reduce the model’s prediction error and improve its prediction accuracy. Meanwhile, compared with model M5 under the prediction scenarios of the two cities, the variant GSPMSTL-M5 achieves improvements of 20.22% and 1.86% in *MAE* and *R*^2^, as well as 25.95% and 3.99%, respectively. This effectively verifies that applying the GSPMSTL decomposition algorithm to PM2.5 time-series prediction can effectively improve the prediction accuracy and performance stability of the model. In addition, compared with M4, model M5 obtains *R*^2^ indicator improvements of 5.54% and 3.11% in the two cities, accompanied by *RMSE* indicator improvements of 30.31% and 13.30%, respectively. This confirms the feasibility of enhancing PM2.5 feature information extraction by introducing CNN on the basis of the LSTM model architecture for model performance improvement. In summary, there are no redundant modules inside the target model M8 proposed in this study, and each component exerts a positive driving effect on the improvement of the model’s prediction performance.

## Discusstion

### Analysis of model complexity

Model complexity can reflect the computational overhead and resource- consumption level of deep-learning models, and serves as an important basis for measuring the practical deployment feasibility of models. To this end, this study introduces two quantitative indicators, namely the total number of trainable parameters (TP) and the floating-point operations (FLOPs) for single-sample forward inference. Combined with the prediction performance of the model on the entire-period test-set of the two cities, comparative complexity analysis of the model is carried out from two aspects: model-architecture complexity and decomposition-algorithm complexity. The results are shown in Tables 9 and 10, respectively.

**Table 10:** Decomposition-algorithm-oriented Complexity Analysis.

Model	Algorithm	Time complexity	Space complexity
M6	EMD	O(N)	O(N)
M7	VMD	O(N × logN)	O(N)
GSPMSTL-M5	GSPMSTL	O(N)	O(N)

**Table 9:** Model-architecture-oriented Complexity Analysis.

Model	Complexity metrics		Guangzhou		Xianyang	
	TP	FLOPs	*R* ^2^	*MAE*	*R* ^2^	*MAE*
M1	41.3470K	85.3750K	0.8660	3.2652	0.8933	8.6829
M2	10.1130K	1.2180M	0.9024	2.6769	0.8722	8.9818
M3	12.4170K	24.7690K	0.8305	3.6188	0.8456	8.3093
M4	20.8010K	41.8250K	0.9322	2.1397	0.8793	8.6421
M5	36.7370K	140.8970K	0.9511	1.7951	0.9269	5.8993

Note: TP is the total number of trainable parameters and FLOPs is the total number of floating-point operations of the model in a single forward inference.

As can be observed from Table 9, among the compared baseline models, model M5 exhibits relatively better prediction-performance under diverse prediction scenarios. Although its TP and FLOPs indicators are generally higher than those of other models, the overall growth amplitude is within a reasonable range, and the gain in prediction performance matches the increment of model complexity. The results demonstrate that compared with other heterogeneous baseline models, the CNN-LSTM deep-learning architecture can achieve a better benign balance between prediction performance and complexity overhead. It can be seen from Table 10 that the GSPMSTL decomposition strategy has linear-order time complexity, which is at the same magnitude as the EMD decomposition algorithm and superior to the linear-logarithmic order of VMD. In terms of space complexity, all three decomposition algorithms are of linear-order complexity. Combined with the analysis of Table 7 and Fig 12, compared with the decomposition-based models M6 and M7, the introduction of the GSPMSTL decomposition strategy will not lead to a significant increase in overall model complexity. Meanwhile, it can more effectively improve the comprehensive prediction performance of the hybrid model, which further verifies the rationality of applying the GSPMSTL decomposition strategy to PM2.5 time-series modeling.

In summary, although the target model M8 is slightly higher than some baseline models in terms of model-complexity indicators, such growth is generally within a controllable range, and its prediction accuracy and generalization performance can be effectively enhanced. Meanwhile, it confirms the rationality and effectiveness of the design strategy of “obtaining prediction-performance gains through moderately increasing the computational overhead of the model”.

### Comparison with existing studies

We screen relevant published studies in recent years whose prediction horizons, data time scales and other settings are consistent with this study, and conduct horizontal performance comparative analysis together with model M8 to further verify the prediction performance of the proposed model. It should be noted that there still exist certain differences among different studies in terms of data collection periods and model operating environments. Therefore, only qualitative comparative analysis is carried out, and accurate quantitative evaluation for the model-performance discrepancies from different studies cannot be implemented. The detailed results are presented in Table 11.

**Table 11:** Performance comparison with previous studies.

Source	Model	Time step	Location	*R* ^2^	*MAE*
Zhang et al.,2020 [45]	NARX	H/M/T + 6	Xianyang	0.9293	—
		H/M/T + 12		0.9068	—
Cheng et al.,2026 [46]	OVMD-TCN-BiLSTM-Attention	H/S/T + 1	Guangzhou	0.9800	1.4420
		H/S/T + 1	Shenzhen	0.9789	1.3300
Wang et al.,2025 [47]	MSTTNET	H/S/T + 1	Guangzhou	0.9135	5.6300
		H/S/T + 1	Shanghai	0.9152	6.8200
		H/S/T + 1	Beijing	0.9393	11.0600
Ours	FATA-GSPMSTL-CNN-LSTM	H/M/T + 6	Xianyang	0.9794	4.0410
		H/M/T + 12	Xianyang	0.9578	5.7260
		H/S/T + 1	Guangzhou	0.9845	1.0071

Notes: H/S/T + X is the X-step direct multi-hour prediction mode based on hourly time series data, H/M/T + Y is the Y-step direct multi-hour prediction mode based on hourly time series data.

As can be seen from the table, for the prediction scenario of Xianyang, under the continuous prediction conditions of H/M/T + 6 and H/M/T + 12, model M8 improves the *R*^2^ indicator by 5.39% and 5.62%, respectively, compared with the NARX model proposed by Zhang et al. For the prediction scenario of Guangzhou, the OVMD-TCN-BiLSTM-Attention model proposed by Cheng et al. achieves better performance than the NSTTNET model proposed by Wang et al. under the single-step prediction condition of H/S/T + 1, with *R*^2^ and *MAE* values of 0.9800 and 1.4420, respectively. By comparison, the hybrid model M8 proposed in this study obtains a 0.46% improvement in *R*^2^ and a 30.16% reduction in *MAE* relative to the OVMD-TCN-BiLSTM-Attention model. The above results indicate that the collaborative design which organically combines the FATA, the GSPMSTL decomposition algorithm and the CNN-LSTM hybrid model possesses certain advantages over the models proposed in existing studies for cross-scenario prediction tasks.

### Assessment of this work and future prospects

There exists obvious spatial heterogeneity across different regions in terms of PM2.5 seasonal variation patterns, pollutant emission structures and meteorological background conditions. Combined with the practical performance of the target model in Guangzhou and Xianyang, the proposed model can perform adaptive decomposition for local PM2.5 time-series data via the GSPMSTL decomposition strategy, and adopt the FATA to carry out adaptive fine-tuning for internal parameters of the CNN-LSTM model. For this reason, it possesses favorable cross-regional transfer capability compared with other baseline models.

Despite the relatively competitive predictive performance of the target model, its generalization performance exhibits moderate variations across forecasting scenarios under the influences of PM2.5 concentration levels, fluctuation amplitudes, geographic locations and extreme climatic conditions. Meanwhile, subject to performance decay with increasing prediction steps, there remains considerable room for improvement for the target model when conducting long-term PM2.5 forecasting tasks at weekly, monthly or even annual scales. Furthermore, limited by computational and time costs, although the selected study sites exhibit sound regional representativeness, PM2.5 time-series modeling for special forecasting scenarios such as plateau and island regions still requires further work to verify the model’s feasibility for applications in these areas.

Considering that potentially valuable information remains to be explored within the residual components obtained from seasonal-trend decomposition [48], follow-up research can further construct a secondary decomposition mechanism based on GSPMSTL and implement re-decomposition targeting residual terms. This measure can further strengthen the decomposition algorithm’s capacity to interpret complex feature patterns embedded in time-series. In addition, given that the model shows different modeling capacities for individual modal components, a parallel multi-channel data-flow execution architecture can be established to realize adaptive modeling for diverse modal components, so as to further boost the prediction accuracy and generalization capability of the model.

## Conclusion

This study proposes a hybrid prediction model for PM2.5, named FATA- GSPMSTL-CNN-LSTM. Based on experimental verification and result analysis, the following conclusions are drawn:

(1) The RFECV-Pearson feature selection strategy can effectively extract features with strong linear and nonlinear correlations with PM2.5, avoiding the omission of important features that may occur in single screening mechanisms.
(2) The proposed GSPMSTL adaptive decomposition algorithm effectively alleviates the poor decomposition quality caused by manually specified periodic factors and inflexible selection of seasonal smoothing window parameters in traditional seasonal decomposition methods, thereby improving model prediction performance from the data input level.
(3) Automatic hyperparameter optimization based on the FATA can effectively enhance the prediction accuracy of the model.
(4) In the prediction tasks of Guangzhou and Xianyang, the FATA-GSPMSTL- CNN-LSTM model achieves the best prediction accuracy and exhibits superior accuracy, stability, and generalization ability, which can provide support for air quality early warning and pollution control.
(5) For PM2.5 time-series forecasting scenarios with high concentrations and strong fluctuations, the target model still leaves room for optimization. Future research can further improve its predictive performance from two perspectives: constructing a secondary decomposition mechanism and optimizing the model architecture. In addition, the idea of improving model predictive performance by optimizing data quality at the model input end can provide a reference for subsequent time-series forecasting research.

## Supporting information

S1 Fig Model convergence results in the first repeated experiment. (TIF)

S2 Fig Model convergence results in the second repeated experiment. (TIF)

S3 Fig Model convergence results in the third repeated experiment. (TIF)

S4 Fig Model convergence results in the fourth repeated experiment. (TIF)

S5 Fig Model convergence results in the fifth repeated experiment. (TIF)

S6 Fig Performance regression comparison of various models. (TIF)

S1 Table Pseudocode of MSTL decomposition. (DOCX)

S2 Table Pseudocode of FATA. (DOCX)

S3 Table Initial parameter settings for MSTL decomposition. (DOCX)

S4 Table Hyper-parameter configurations of partial baseline models. (DOCX)

S5 Table Results of model significance statistical test in the heating season. (DOCX)

S6 Table Results of model significance statistical test in the non-heating season. (DOCX)
