# Supplementary material for: A novel deep learning prediction method for PM2.5 integrating improved MSTL decomposition and FATA optimization algorithm
Source: PLoS One. 2026 Sep 15;21(9):e0358271. doi: 10.1371/journal.pone.0358271 (PMC13577383; doi:10.1371/journal.pone.0358271)
Supplement: Supplementary file 1 [file pone.0358271.s007.docx]

**S1 Table. Pseudocode of MSTL decomposition**

| Algorithm 1: MSTL decomposition | |
| --- | --- |
| 1: | **Procedure** **MSTL**(*X*, *iterate*, *λ*, *s*.win[], ...): |
| 2: | **If** *X* is multi-seasonal **then:** |
| 3: | seas.ids[] ⬅ attributes(*X*); |
| 4: | *k* ⬅ len(*X*); |
| 5: | seas.ids ⬅ seas.ids[seas.ids ＜ *k* / 2]; |
| 6: | seas.ids ⬅ sort(seas.ids, dec = *F*); |
| 7: | **Else if** *X* is single-seasonal **then:** |
| 8: | seas.ids[] ⬅ frequency(*X*); |
| 9: | iterate ⬅ 1; |
| 10: | **End if** |
| 11: | *X* ⬅ **na.interp**(*X*, *λ*); |
| 12: | *X* ⬅ **BoxCox**(*X*, *λ*); |
| 13: | **If** seas.ids[1] ＞ 1 **then:** |
| 14: | seasonality ⬅ list(rep(0, len(seas.ids))); |
| 15: | deseas ⬅ *X*; |
| 16: | **For** *j* in 1 to iterate **do:** |
| 17: | **For** *i* in 1 to len(seas.ids) **do:** |
| 18: | deseas ⬅ deseas + seasonality[[i]]; |
| 19: | fit ⬅ **STL**(ts(deseas, frequency = seas.ids[i]), *s*.window = *s*.win[i], ...); |
| 20: | seasonality[[i]] ⬅ msts(seasonal(fit)); |
| 21: | deseas ⬅ deseas - seasonality[[i]]; |
| 22: | **End For** |
| 23: | **End For** |
| 24: | trend ⬅ msts(trendcycle(fit)); |
| 25: | **Else:** |
| 26: | seas.ids ⬅ NULL; |
| 27: | deseas ⬅ *X*; |
| 28: | trend ⬅ ts(**SUPSMU**(X)); |
| 29: | **End If** |
| 30: | remainder ⬅ deseas - trend; |
| 31: | **return** [trend, remainder, seasonality]; |
| 32: | **End procedure** |
