# Supplementary material for: A novel deep learning prediction method for PM2.5 integrating improved MSTL decomposition and FATA optimization algorithm
Source: PLoS One. 2026 Sep 15;21(9):e0358271. doi: 10.1371/journal.pone.0358271 (PMC13577383; doi:10.1371/journal.pone.0358271)
Supplement: Supplementary file 2 [file pone.0358271.s008.docx]

**S2 Table. Pseudocode of FATA**

| Algorithm 2: FATA | |
| --- | --- |
| 1: | **Input**: parameters *n*, *d,* *MaxFEs*; |
| 2: | **Output**: best Individual; |
| 3: | Initialization parameters *Para*_1_, *Para*_2_, *α*； |
| 4: | Initialize a population x of size *n*; |
| 5: | Calculate the fitness of each individual; |
| 6: | **While** (*FEs* ≤ *MaxFEs*) |
| 7: | update best fitness, *x*_best_; |
| 8: | Calculate weights *P*; |
| 9: | Calculate *Para*_1_ and *Para*_2_; |
| 10: | **For** i = 1 : *n* |
| 11: | Execute Algorithm 1 to realize **the mirage light filtering principle**; |
| 12: | **If** *rand* ＞ *P* |
| 13: | the light population performs to initialize the population randomly; |
| 14: | **Else** |
| 15: | **If** *rand* ＜ *q* |
| 16: | Update the individual *x*_i_ according to **the first half light refraction strategy**; |
| 17: | **Else** |
| 18: | Update the individual *x*_i_ according to **the second half light refraction strategy**; |
| 19: | Update the individual *x*_i_ according to **the light total internal refraction strategy**; |
| 20: | **End If** |
| 21: | **End If** |
| 22: | **End For** |
| 23: | *t* = *t* + 1; |
| 24: | **End While** |
| 25: | **Return** the best individual *x*_best_; |
