# Supplementary material for: A novel deep learning prediction method for PM2.5 integrating improved MSTL decomposition and FATA optimization algorithm
Source: PLoS One. 2026 Sep 15;21(9):e0358271. doi: 10.1371/journal.pone.0358271 (PMC13577383; doi:10.1371/journal.pone.0358271)
Supplement: Supplementary file 3 [file pone.0358271.s009.docx]

**S3 Table. Initial parameter settings for MSTL decomposition**

| Index | Guangzhou | Xianyang |
| --- | --- | --- |
| iterate | 5 | 5 |
| p | 6、8、12、24 | 12、24 |
| n_s_ | 5、9、11、19 | 7、23 |
| n_t_ | 21 | 39 |
| degree | 2 | 2 |
