# Supplementary material for: A novel deep learning prediction method for PM2.5 integrating improved MSTL decomposition and FATA optimization algorithm
Source: PLoS One. 2026 Sep 15;21(9):e0358271. doi: 10.1371/journal.pone.0358271 (PMC13577383; doi:10.1371/journal.pone.0358271)
Supplement: Supplementary file 4 [file pone.0358271.s010.docx]

**S4 Table. Hyper‑parameter configurations of partial baseline models**

| **City** | **Model** | **Parameter type** | **Search range** | **Optimal value** |
| --- | --- | --- | --- | --- |
| Guangzhou | M1 | Hidden dimension | {32, 64, 128} | 64 |
|  |  | Learning rate | {0.0001, 0.0005, 0.001} | 0.001 |
|  |  | Optimizer type | {Adam, Adagrad} | Adam |
|  |  | GRU Layers | {1, 2} | 1 |
|  | M2 | *d_model_* | {32, 64, 128} | 64 |
|  |  | Learning rate | {0.0001, 0.0005, 0.001} | 0.0005 |
|  |  | Optimizer type | {Adam, Adagrad} | Adam |
|  | M3 | Hidden dimension | {32, 64, 128} | 64 |
|  |  | Learning rate | {0.0001, 0.0005, 0.001} | 0.0001 |
|  |  | Optimizer type | {Adam, Adagrad} | Adam |
|  | M4 | Hidden dimension | {32, 64, 128} | 32 |
|  |  | Learning rate | {0.0001, 0.0005, 0.001} | 0.001 |
|  |  | Optimizer type | {Adam, Adagrad} | Adagrad |
|  |  | GRU Layers | {1, 2} | 2 |
| Xianyang | M1 | Hidden dimension | {32, 64, 128} | 32 |
|  |  | Learning rate | {0.0001, 0.0005, 0.001} | 0.001 |
|  |  | Optimizer type | {Adam, Adagrad} | Adagrad |
|  |  | GRU Layers | {1, 2} | 1 |
|  | M2 | d_model_ | {32, 64, 128} | 32 |
|  |  | Learning rate | {0.0001, 0.0005, 0.001} | 0.001 |
|  |  | Optimizer type | {Adam, Adagrad} | Adam |
|  | M3 | Hidden dimension | {32, 64, 128} | 64 |
|  |  | Learning rate | {0.0001, 0.0005, 0.001} | 0.0005 |
|  |  | Optimizer type | {Adam, Adagrad} | Adam |
|  | M4 | Hidden dimension | {32, 64, 128} | 32 |
|  |  | Learning rate | {0.0001, 0.0005, 0.001} | 0.0005 |
|  |  | Optimizer type | {Adam, Adagrad} | Adam |
|  |  | GRU Layers | {1, 2} | 1 |

Note: *d_model_* is the model dimension of TimesNet, representing the dimension of the latent embedding space output from the embedding projection layer.
