# Supplementary material for: A novel deep learning prediction method for PM2.5 integrating improved MSTL decomposition and FATA optimization algorithm
Source: PLoS One. 2026 Sep 15;21(9):e0358271. doi: 10.1371/journal.pone.0358271 (PMC13577383; doi:10.1371/journal.pone.0358271)
Supplement: Supplementary file 5 [file pone.0358271.s011.docx]

S5 Table. Results of model significance statistical test in the heating season

| Target model | Comparison model | Guangzhou | | Xianyang | |
| --- | --- | --- | --- | --- | --- |
|  |  | DM value | P value | DM value | P value |
| M8 | M1 | 30.9448 | 0.0000 | 25.7326 | 0.0000 |
|  | M2 | 22.2384 | 0.0000 | 8.5873 | 0.0000 |
|  | M3 | 42.6227 | 0.0000 | 30.4442 | 0.0000 |
|  | M4 | 31.1823 | 0.0000 | 23.3960 | 0.0000 |
|  | M5 | 18.7676 | 0.0000 | 13.5478 | 0.0000 |
|  | M6 | 25.6140 | 0.0000 | 19.2391 | 0.0000 |
|  | M7 | 21.9435 | 0.0000 | 4.4452 | 0.0000 |
