# Supplementary material for: A novel deep learning prediction method for PM2.5 integrating improved MSTL decomposition and FATA optimization algorithm
Source: PLoS One. 2026 Sep 15;21(9):e0358271. doi: 10.1371/journal.pone.0358271 (PMC13577383; doi:10.1371/journal.pone.0358271)
Supplement: Supplementary file 6 [file pone.0358271.s012.docx]

S6 Table. Results of model significance statistical test in the non-heating season

| Target model | Comparison model | Guangzhou | | Xianyang | |
| --- | --- | --- | --- | --- | --- |
|  |  | DM value | P value | DM value | P value |
| M8 | M1 | 28.3812 | 0.0000 | 14.3236 | 0.0000 |
|  | M2 | 17.7712 | 0.0000 | 6.1905 | 0.0000 |
|  | M3 | 28.5065 | 0.0000 | 18.0538 | 0.0000 |
|  | M4 | 26.7647 | 0.0000 | 15.4154 | 0.0000 |
|  | M5 | 19.0752 | 0.0000 | 11.6016 | 0.0000 |
|  | M6 | 22.8984 | 0.0000 | 16.6655 | 0.0000 |
|  | M7 | 20.2331 | 0.0000 | 3.3492 | 0.0005 |
